# Polyurea Aerogels: Synthesis, Material Properties, and Applications

**DOI:** 10.3390/polym14050969

**Published:** 2022-02-28

**Authors:** Nicholas Leventis

**Affiliations:** Aspen Aerogels, Inc. 30 Forbes Road, Bldg B, Northborough, MA 01532, USA; nleventis@aerogel.com; Tel.: +1-508-847-1792

**Keywords:** polyurea, aerogel, isocyanate, amine, water, mineral acid, structure–property relationships, nanomorphology, *K*-index

## Abstract

Polyurea is an isocyanate derivative, and comprises the basis for a well-established class of polymeric aerogels. Polyurea aerogels are prepared either via reaction of multifunctional isocyanates with multifunctional amines, via reaction of multifunctional isocyanates and water, or via reaction of multifunctional isocyanates and mineral acids. The first method is the established one for the synthesis of polyurea, the third is a relatively new method that yields polyurea doped with metal oxides in one step, while the reaction of isocyanates with water has become the most popular route to polyurea aerogels. The intense interest in polyurea aerogels can be attributed in part to the low cost of the starting materials—especially via the water method—in part to the extremely broad array of nanostructural morphologies that allow study of the nanostructure of gels as a function of synthetic conditions, and in part to the broad array of functional properties that can be achieved even within a single chemical composition by simply adjusting the synthetic parameters. In addition, polyurea aerogels based on aromatic isocyanates are typically carbonizable materials, making them highly competitive alternatives to phenolic aerogels as precursors of carbon aerogels. Several types of polyurea aerogels are already at different stages of commercialization. This article is a comprehensive review of all polyurea-based aerogels, including polyurea-crosslinked oxide and biopolymer aerogels, from a fundamental nanostructure–material properties perspective, as well as from an application perspective in thermal and acoustic insulation, oil adsorption, ballistic protection, and environmental cleanup.

## 1. Introduction

The term “aerogel” describes a certain class of low-density solid materials with a high open porosity. Aerogels can be considered to be a subclass of the much broader domain of porous materials; however, they are distinguished from, for example, blown foams, because they are prepared in a completely different manner—namely, by drying wet gels in a way that preserves nearly all of their volume in the final dry form [1,2]. This definition not only distinguishes aerogels from other porous materials, but also expands their list to include porous carbon and ceramic materials that come from sol–gel-derived wet gels dried in that particular manner. The first aerogels were reported in 1931 [3], and they were prepared in order to study the solid frameworks of wet gels. However, owing to their unique material properties, aerogels soon thereafter took on a life of their own, independent of fundamental studies of colloidal systems.

Within the group of sol–gel-derived porous materials, aerogels occupy the low-density extreme of the continuum that extends from xerogels, through ambigels, to aerogels, and depends on the degree of shrinkage of the wet gels upon drying [4,5,6]. Xerogels and ambigels are obtained by evaporation of the pore-filling solvent of a wet gel under ambient pressure. For xerogels, the evaporating solvent is usually the gelation solvent itself. For ambigels, prior to drying, the gelation solvent is exchanged with a low-surface-tension solvent (typically pentane, hexane, or cyclohexane), resulting in much lower density materials [5,6]. Aerogels are typically obtained by using a pressure vessel to convert the pore-filling solvent of a wet gel into a supercritical fluid (SCF), which is then vented off like a gas [4]. This process is referred to as supercritical drying, and is based on the fact that suddenly converting the entire amount of the pore-filling solvent into a gas-like state practically eliminates surface tension (capillary) stresses on the solid network. Any small degree of shrinkage that may still be observed is attributed primarily to molecular-level interactions of the skeletal network with itself when the solvent is removed from the pores [7]. It should be noted further that the aforementioned drying methods do not define the materials, but rather describe how such materials are most commonly made; indeed, other drying methods—for example, freeze-drying—can yield materials with similar properties to those obtained via supercritical drying, or on other occasions the skeletal network is either strong enough or elastic enough that evaporative drying akin to xerogelling or ambigelling may yield aerogels. 

In terms of chemical identity, the skeletal framework of aerogels can be inorganic, organic, or a combination of the two. Inorganic aerogels are based on oxides, chalcogenides, metals, ceramics (e.g., carbides, nitrides), and carbon. Organic aerogels are based on synthetic or natural polymers. Several examples of the latter class (e.g., cellulose, nitrocellulose, gelatin, agar, egg albumin, and rubber aerogels) were already described in the pioneering aerogel publications in the early 1930s [3,8]. However, subsequent focus on oxide aerogels—and especially silica—diverted attention away from polymeric aerogels until the introduction of aerogels based on phenolic resins—particularly those based on the condensation of resorcinol with formaldehyde in the late 1980s. From that point on, and for almost 20 years, resorcinol–formaldehyde aerogels became practically synonymous with organic aerogels. This situation started to change as a handful of isolated publications and patents in the late 1990s and through the 2000s planted the seeds for the rapid development of polymeric aerogels that occurred in the last decade. Those early works included the first examples of aerogels based on polyurethanes [9], polyimides [10], polydicyclopentadiene [11], polyurea and other polyisocyanate based aerogels [12], polybenzoxazines [13], conducting polymers [14] and, importantly, a new class of organic–inorganic nanocomposite aerogels referred to as polymer-crosslinked aerogels [15]. Polymer-crosslinked aerogels showed that aerogels are not necessarily fragile materials, and catalyzed a shift of attention toward polymeric aerogels, including those based on isocyanate chemistry, and in particular those based on polyurea, which is the subject of this chapter. 

In the first implementations of the polymer-crosslinked aerogel technology, a preformed inorganic 3D network of sol–gel-derived inorganic nanoparticles (primarily silica, but including several other oxide wet gels [15,16,17]) served as a template for the deposition of a conformal, nanothin polymer coating over the entire skeletal network of the wet gel. The morphology of the network did not change, but the resulting nanocomposite aerogels were extremely strong mechanically. At this point, it was reasoned that since the dramatic improvement in strength was brought about by the crosslinking polymer, if the polymer itself could be rendered into an analogous form comprising similarly sized nanoparticles connected as in the polymer-crosslinked oxide aerogels, the resulting all-polymer aerogel should exhibit similar mechanical properties. Early polymer-crosslinked silica aerogels were obtained via the reaction of various triisocyanates with the hydroxyl groups on the surface of silica. However, the bulk of the polymer connecting skeletal nanoparticles together in the resulting nanocomposites was identified as polyurea arising from the reaction of isocyanate with strongly adsorbed gelation water remaining on the nanoparticles of the silica wet-gel framework [18,19]. Thus, based on this chemical–topological rationale, a new class of polyurea aerogels was synthesized via reaction of triisocyanates with water, and the immediate property of interest was the mechanical strength [20]. As it turned out, these isocyanate/water polyurea aerogels exhibit a tremendously versatile skeletal nanostructure depending on the synthetic conditions (e.g., solvent, monomer, and catalyst concentrations). Thus, having run a full circle, aerogels—and especially those of polyurea—again serve as proxies, or as a powerful tool, for the study of the structure–property relationships in wet gels, in the spirit of Kistler’s pioneering work almost 90 years ago. At this point, it is appropriate to mention that these structure–property studies are assisted tremendously by the modern analytical methodology that was not available in Kistler’s time. Modern analytical techniques allow detailed characterization of materials and structure–property correlation from the molecular to macroscopic through the nano-scale regimes, in a continuous and often overlapping fashion. The gelation process itself is followed by multinuclear liquid-phase NMR, with light, X-ray, and neutron scattering, as well as rheology. At the molecular level, classic techniques for the analysis of the resulting aerogels include elemental analysis (via classic combustion techniques, but also with techniques ranging from energy-dispersive spectroscopy (EDS) all the way to neutron activation analysis), infrared and X-ray photoelectron spectroscopy (XPS), solid-state NMR, and X-ray diffraction (XRD). Relevant to the nanoscopic structure of aerogels is the particle size and assembly, as well as the pore volume and structure. The usual methods to probe the solid framework include SEM, TEM, as well as X-ray, and neutron-scattering techniques. The pore volume and structure are evaluated from the micro- to meso- to macro pore size regimes via gas sorption methods (typically N_2_ at 77 K and CO_2_ at 0 °C). A non-distractive “look” inside macroscopic aerogel monoliths is usually achieved via X-ray tomography. Mechanical properties such as the elastic modulus, the yield and ultimate strength, and the energy absorption are the common properties of interest of large aerogel samples (“large” here means from a few microns to a few tens of centimeters). Typically, mechanical properties are recorded under compression, less frequently under tension, and when no large, regular-shaped pieces are available, nanoindentation has been a technique of choice. Looking for patterns and trends, all types of studies are usually conducted with a series of samples with the same chemical composition, but different bulk densities. The reader is directed to references 20, 39, and 83, which each combine more than 15 different techniques at a time for the characterization of a single type of aerogel. 

The material collected for this article is organized as follows: Section 2 presents an overview of the chemistry of the isocyanate group pertinent to the formation of polyurea aerogels. Section 3 continues with a brief discussion of the relationship between nanostructure and the gelation process, and of methods to form nanostructured polyurea aerogels into useful shapes (monoliths, beads etc.). Section 4 provides an up-to-date literature review of polyurea aerogels; this section follows a parallel organization with Section 2, whereby literature reports are grouped according to the controlling chemistry. Section 4 also includes recent advances in polymer aerogel composites, such as robust polyurea-crosslinked oxide and biopolymer aerogels. Applications are reviewed together with the corresponding materials in the respective sections. Finally, Section 5 presents some conclusions, and summarizes the most important lessons learned from polyurea aerogels, while providing a glimpse into important implications those findings might have in the future. 

## 2. The Chemistry of Polyurea Aerogels

### 2.1. The Chemistry of the Isocyanate Group

**Figure 1:**
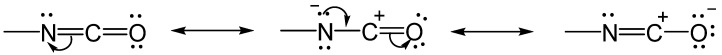
Resonance within the isocyanate (–N=C=O) group.

**Figure 2:**
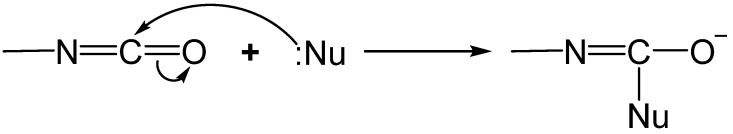
Addition of a nucleophile (:Nu) to the isocyanate group.

The carbon atom of the isocyanate group, –N=C=O, is in the 4+ oxidation state, and is therefore oxidatively stable. The reactivity of the isocyanate group is governed by the electron-withdrawing effects of the oxygen and nitrogen atoms (Figure 1), leaving the carbonyl carbon with an enhanced partial positive charge and, thus, susceptible to nucleophilic attack (Figure 2). 

Table 1 compares the uncatalyzed relative reactivity of some common N- and O-based active hydrogen nucleophiles toward the –N=C=O group, as shown in Figure 2 [21]. “Active-hydrogen” refers to nucleophiles that carry all atoms necessary to form stable neutral species; for instance, ROH—an alcohol—is an active hydrogen nucleophile because at the end it forms neutral urethane, but the corresponding alkoxide, RO^–^, although more reactive toward -N=C=O, is not considered to be an active hydrogen nucleophile. In the context of polyurea formation, for steric reasons, secondary aliphatic amines—and for that matter, secondary alcohols—although stronger bases than their primary counterparts, react more slowly than the latter. 

**Figure 3:**
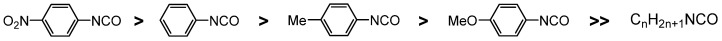
The reactivity of the isocyanate group as a function of substitution on the nitrogen of NCO.

The reactivity of the isocyanate group (–N=C=O) is modulated by the electron-withdrawing or electron-donating ability of the groups attached to N. Aromatic isocyanates are generally more reactive than aliphatic ones [22]. Electron-withdrawing substituents on the aromatic ring enhance the positive charge on the –N=C=O carbon even further and, all other factors being equal (e.g., steric effects), they increase its reactivity toward nucleophiles [23]. Conversely, electron donation due to either resonance (e.g., MeO–) or induction (e.g., CH_3_–) reduces the reactivity of the –N=C=O group. Figure 3 ranks various aromatic and aliphatic isocyanates in order of their relative reactivity [22]. 

**Figure 4:**
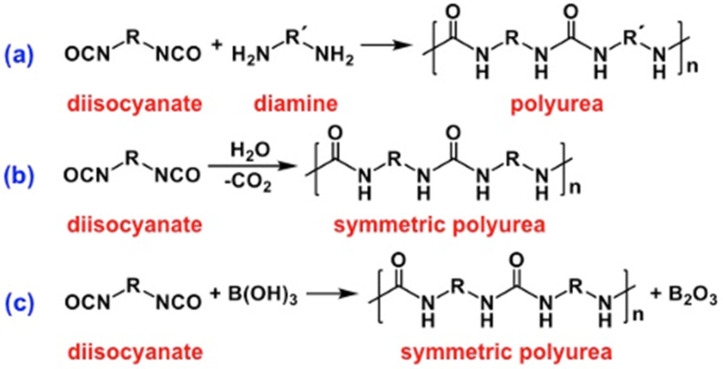
The three routes to polyurea aerogels via reaction of multifunctional isocyanates (exemplified here with a diisocyanate) with (**a**) multifunctional amines, (**b**) water, and (**c**) mineral acids (exemplified here with boric acid).

As noted in Table 1, the fastest reaction of the isocyanate group is with amines to yield ureas. However, as summarized in Figure 4, in addition to this straightforward approach to polyurea aerogels, there are two more methods: via reaction of multifunctional isocyanates with water, and with mineral acids. The following subsections of Section 2 review and compare these three processes.

### 2.2. Formation of Polyurea Aerogels via Reaction of the –N=C=O with Amines

**Figure 5:**
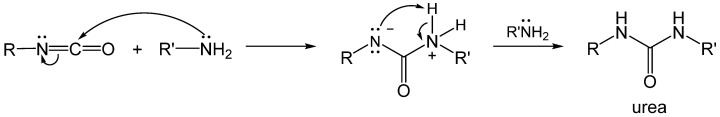
Urea formation via reaction of an isocyanate with an amine.

When the nucleophile in Figure 2 is an amine, the product is a urea (Figure 5). The reaction is exothermic, self-catalytic and, typically, fast. The 1,3-proton transfer tautomerization between the two nitrogens in the second step is catalyzed by another molecule of the amine itself acting as a base. Aromatic amines are weaker nucleophiles than aliphatic amines and, therefore, react more slowly than their aliphatic counterparts [24].

**Figure 6:**
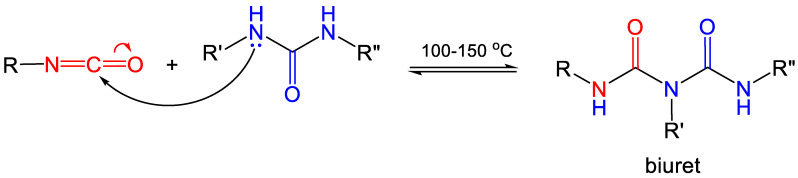
The reaction of isocyanates with ureas to form biurets. The arrows show the initial nucleophilic attack. The overall reaction involves several intermediates. Color coding is used for tracking purposes.

Ureas, in turn, acting as *N*-based nucleophiles, can attack excess isocyanate to yield biurets (Figure 6). However, as noted in Table 1, the nucleophilicity of ureas toward –N=C=O is low, and the reaction takes place at higher temperatures (100–150 °C) [22,25]. Nevertheless, the formation of biurets serves as a crosslinking mechanism for polyurea.

**Figure 7:**
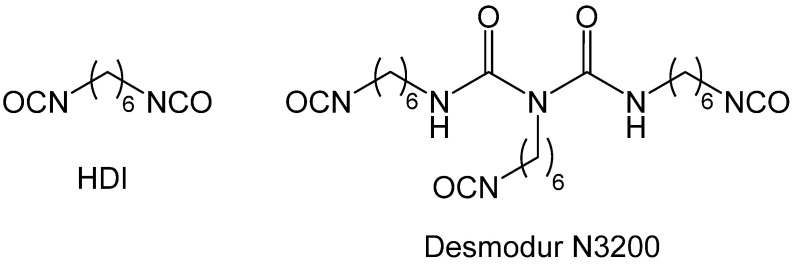
Hexamethylene diisocyanate (HDI) and the primary component of Desmodur N3200 (a biuret derivative of HDI), manufactured by Covestro LLC (Pittsburgh, PA, USA).

Desmodur N3200 from Covestro LLC—a triisocyanate that has been used extensively in the synthesis of polyurea and polyurea-crosslinked oxide aerogels—is a biuret derived from hexamethylene diisocyanate (HDI) (Figure 7) [26]. 

### 2.3. Formation of Polyurea Aerogels via Reaction of –N=C=O with Water

**Figure 8:**
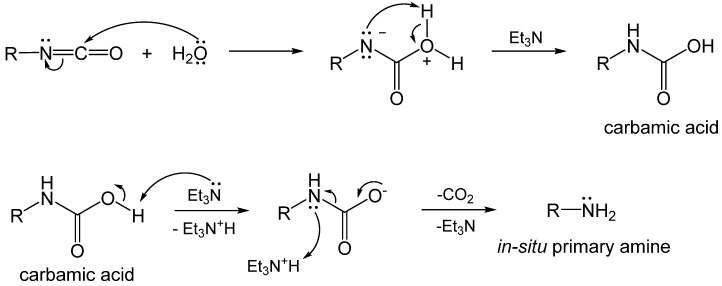
In situ formation of amines from the reaction of isocyanates with water. The 1,3-proton transfer tautomerization of the first step is catalyzed by non-nucleophilic tertiary amines such as Et_3_N. In the second step, Et_3_N first undergoes an acid–base reaction with carbamic acid. The resulting carbamate expels CO_2_, while also taking back a proton from [Et_3_NH]^+^ to form an amine.

Water acting as a nucleophile attacks the isocyanate carbonyl to yield a carbamic acid, which is unstable and decomposes into an amine and carbon dioxide (Figure 8). The hydrolytic reaction leading to the carbamic acid is slow (Table 1), and in practice is usually catalyzed by non-nucleophilic tertiary amines, e.g., triethylamine (Et_3_N). The primary amine formed by decomposition of the carbamic acid reacts rapidly with a second, unreacted isocyanate, as shown in Figure 5, forming a symmetric urea [27,28]. Since the sequence of reactions in Figure 5 and Figure 8 bypasses the use of extraneous amines for the synthesis of ureas, it represents a cost-effective alternative to the process of Figure 5 for synthesizing symmetric polyurea aerogels. By the same token, however, the reaction sequence of Figure 5 and Figure 8 is not an atom-efficient process, as a carbon atom is lost as CO_2_. Therefore, this reaction pathway is not typically used for the synthesis of bulk polyurea. Industrially, a small amount of water is often added deliberately as a foaming agent during preparation of bulk polyurethane foams [29]. When it comes to the synthesis of polymeric aerogels, however, loss of CO_2_ is not a deterrent, because aerogels require only small amounts of monomers, and the cost of the mass loss due to CO_2_ evolution is just a small fraction of the overall cost. Although CO_2_ evolution during polyurea aerogel synthesis can potentially result in unwanted voids in the resulting monolithic parts, in practice this has not been reported as a problem.

**Figure 9:**
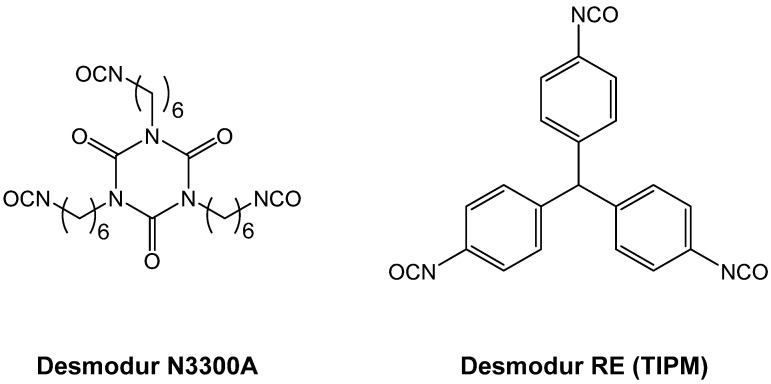
Commercially available triisocyanates (from Covestro LLC) that have been used extensively for making polyurea aerogels via reaction with water. Desmodur N3300A: an isocyanate trimer of hexamethylene diisocyanate (HDI, see Figure 7), supplied as a pure compound. Desmodur RE: Tris(4-isocyanatophenyl)methane, a rigid aromatic triisocyanate, usually abbreviated as TIPM, supplied as an ethyl acetate solution.

In addition to Desmodur N3200 (Figure 7), other multifunctional isocyanates that have been used in polyurea aerogel synthesis include the two triisocyanates shown in Figure 9. 

### 2.4. Formation of Polyurea Aerogels via Reaction of –N=C=O with Mineral Acids

**Figure 10:**
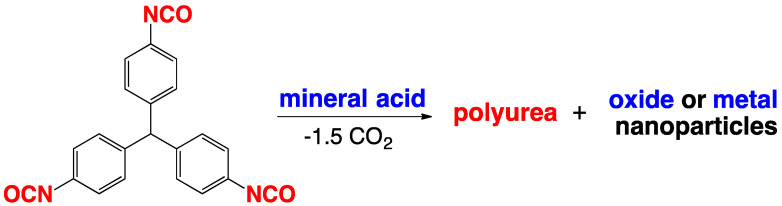
General gelation pathway from reaction of a triisocyanate (Desmodur RE; see Figure 9) with mineral acids.

**Figure 11:**
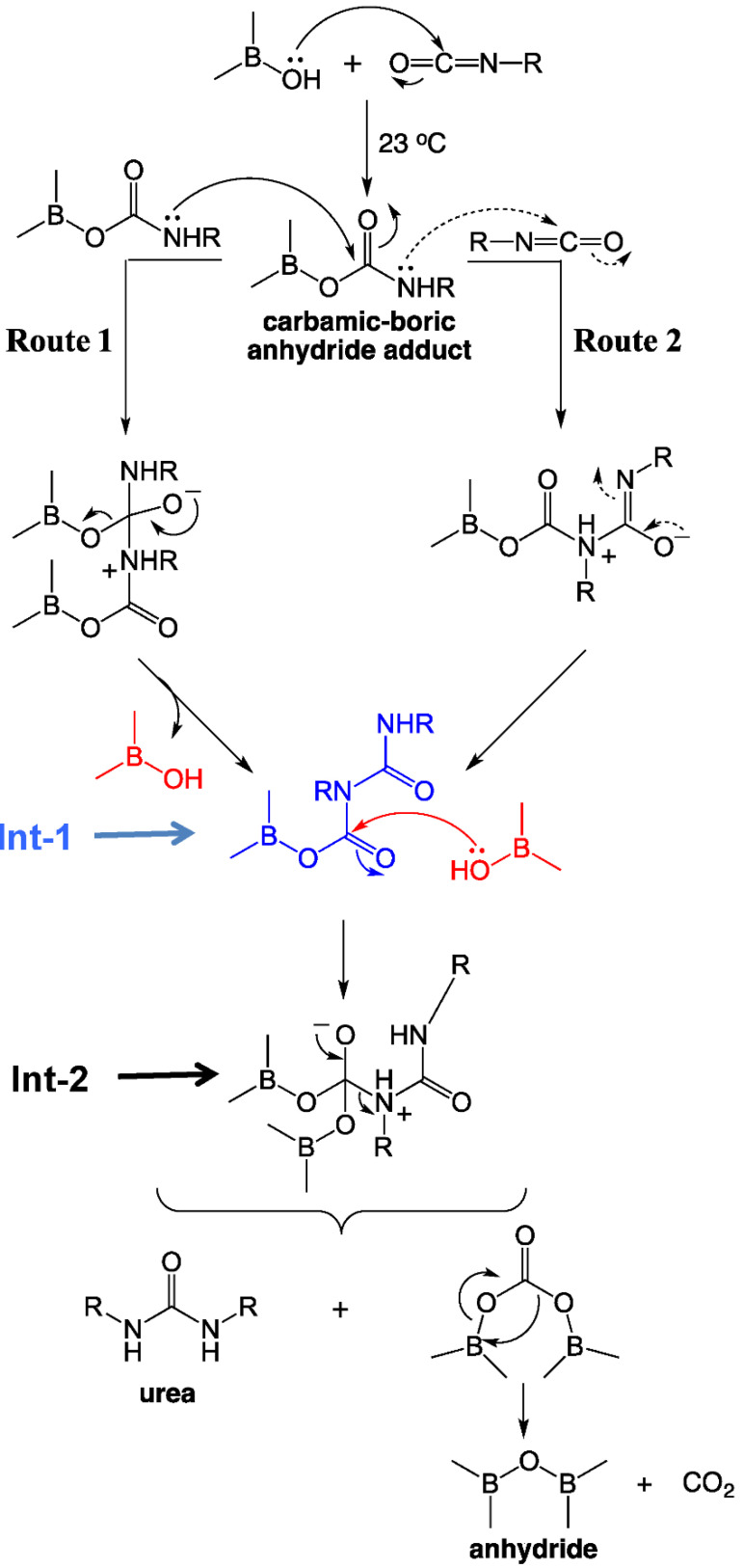
Proposed mechanism for the formation of urea from isocyanate and H_3_BO_3_ (Note, boric acid is used here as a proxy for any of several other possible mineral acids).

The reaction of isocyanates with mineral acids to yield polyurea aerogels was first reported in 2016 [30], and is summarized in the most general terms in Figure 10. This discovery stemmed from an effort to prepare boramic aerogels by reproducing a procedure reported in a 1962 patent [31]. According to that procedure, boramides (i.e., materials with –B–NH– linkages) could be prepared via reaction of isocyanates with boric acid, in analogy to the well-understood reaction of isocyanates with carboxylic acids, yielding amides [32,33]. What was obtained instead was a pure polyurea aerogel. The proposed mechanism starts with the formation of the carbamic–boric anhydride adduct, in analogy to the reaction of –COOH with –N=C=O. The carbamic–boric anhydride adduct can then react with another molecule of the same kind (Figure 11, Route 1), or with an isocyanate (Figure 11, Route 2). The two routes converge to a common intermediate (Figure 11, **Int-1**) that reacts with another molecule of boric acid. The next intermediate (**Int-2**) rearranges itself into urea and boric oxide (B_2_O_3_: the anhydride of boric acid). Overall, the isocyanate/mineral acid system behaved as if it proceeded along an established pathway in the reaction of –COOH and –N=C=O, but stopped at the intermediate urea/anhydride stage. B_2_O_3_ can be removed completely during post-gelation washes, leaving behind a pure polyurea wet gel, which is dried into an aerogel [30]. 

This new pathway to polyurea aerogels is quite general; in addition to H_3_BO_3_, reaction of the same triisocyanate with H_3_PO_4_, H_3_PO_3_, H_2_SeO_3_, H_6_TeO_6_, H_5_IO_6_, and H_3_AuO_3_ always yielded the same polyurea aerogel; however, while the anhydride side product from the reaction with H_3_BO_3_ (that is B_2_O_3_) could be washed off easily from the porous structure of the wet gels, the other oxides were insoluble, and remained as nanodispersed dopants in the final polyurea aerogels, as well as in the corresponding carbon aerogels derived from them by pyrolysis. An interesting exception was gelation with auric acid where, due to the instability of the oxide, both the corresponding polyurea aerogel and the derived carbon aerogel were doped with nanodispersed Au nanoparticles. 

## 3. Translating the Polymerization Chemistry into Aerogels

### 3.1. The Gelation Process and Nanomorphology

The polymerization processes described in Section 2 turn soluble monomers into insoluble polymers that comprise the skeletal framework of the aerogel. The morphology of the skeletal framework of polyurea aerogels can be very diverse, even within the well-defined chemical composition of a specific polymer. This morphological diversity arises from parameters such as the molecular structure of the monomers (e.g., aromatic versus aliphatic, rigid versus flexible, difunctional versus polyfunctional, the functional group density at the monomer molecular level), the solubility properties of the medium (e.g., polarity, ability to develop dispersion forces, and hydrogen bonding), the concentrations of the monomers and the catalyst, and the gelation temperature. 

**Figure 12:**
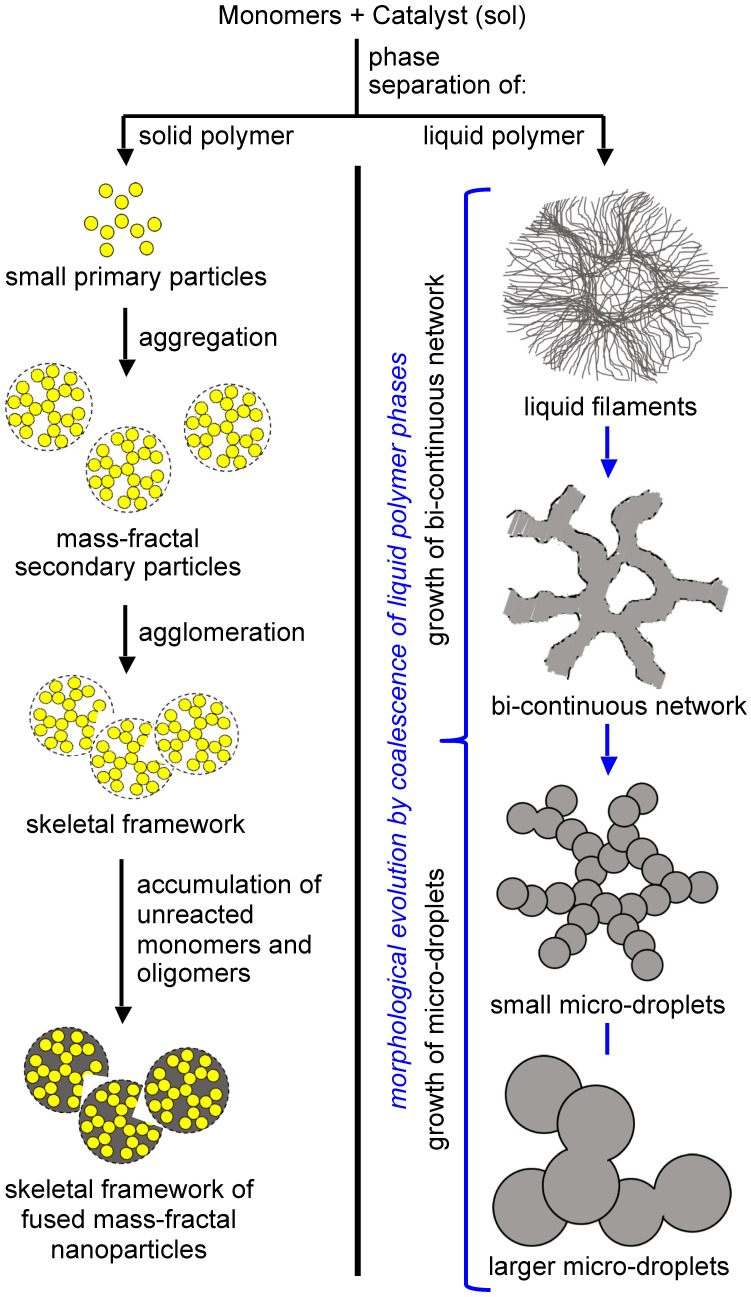
Phase separation and growth processes involved in the formation of the skeletal network of gels.

The formation of an insoluble polymer means phase separation, which can be of either solid primary particles (Figure 12, left branch), or of an oily phase of insoluble oligomers (Figure 12, right branch) [34,35,36]. In the first case, the size of the primary particles decreases as the functionality and functional-group density of the monomers increases, leading quickly to a highly crosslinked, insoluble polymer. This has been demonstrated well with poly(urethane acrylate) aerogels derived from trifunctional versus nine-functional (nonafunctional) star and dendritic monomers, respectively; the two types of monomers, made using the same rigid aromatic triisocyanate core, yielded primary particles with radii equal to 17 nm versus 7 nm, respectively [37,38].

Phase-separated primary particles move randomly in the sol, and upon collision with one another they may become chemically connected via reaction with yet-unreacted surface functional groups inherited from the monomers. If the reaction is fast (and, thus, the sticking probability for each collision is high), the kinetics of network growth are limited only by diffusion, and the process is referred to as diffusion-limited aggregation. Such aggregates are mass fractal objects [36], and their size is limited by the fact that their density falls rapidly with distance from the nucleation point at their center; this is to say that beyond a certain size there is not enough mass in their outer “layers” to support further growth. These fractal aggregates of primary particles are referred to as secondary particles. On geometric grounds, the pore sizes within secondary particles are in the same order as the primary particles, and typically they fall in the mesopore range (2–50 nm). Secondary particles also randomly walk, albeit more slowly, eventually being connected to one another via chemical bonding to form higher aggregates. The liquid sol turns into a solid gel once secondary particles, or higher aggregates, have formed a continuous interconnected three-dimensional path spanning the entire volume of the liquid within a mold. At that point, assuming that all monomers have been consumed, the skeletal network consists of interconnected secondary particles or higher aggregates. Experimentally, primary particles are identified, and their size is estimated using small-angle X-ray or neutron-scattering data (SAXS or SANS, respectively); alternatively, the primary particle size is calculated from skeletal density and N_2_ sorption data via *r* = 3/(*ρ*_s_ × *σ*), where *r* is the particle radius, *ρ*_s_ is the skeletal density from He pycnometry; and *σ* is the BET surface area from N_2_ sorption porosimetry. The primary particle size obtained via the two methods should be roughly equal. This situation is very common with oxide aerogels, and is also encountered with several of the polyurea aerogels reviewed in this article. If unreacted monomers or soluble oligomers remain in the pores after gelation, these species eventually find their way onto the protoskeletal framework, where they can accumulate via reaction with still-live functional groups on the surface of the framework. Eventually, accumulation of monomers and/or oligomers fills the void space within the secondary particles and, experimentally, the particle size calculated from skeletal density and N_2_ sorption data reflects a size closer to that of the secondary particles as determined via SAXS or SANS. Under SEM, these structures appear as if they consist of random assemblies of particles, with a layer of polymer cast over the entire network [37]. The topography of such an arrangement actually resembles that of polymer-crosslinked oxide aerogels, in which silica primary particles are embedded within a crosslinker-derived polymer that fills the space within the aerogel’s secondary particles [39,40]. Comparisons of particle sizes determined from skeletal density/N_2_ sorption data and SAXS have proven useful in understanding the growth mechanisms underlying the formation of polymeric aerogels in general. 

An interesting variation of the theme described in the previous paragraph is encountered when, within a series of aerogel samples of variable density, the particle size determined from skeletal density/N_2_ sorption data matches the primary particle size from SAXS for high-density aerogels, but diverges—sometimes by orders of magnitude—at lower densities. This type of density-dependent trend is attributed to a sol-concentration-dependent gelation mechanism, and is typically accompanied by obvious morphological changes in SEM and large changes in the BET surface area [41]. 

Phase separation of oligomers as an oily liquid (Figure 12, right branch) occurs with large flexible monomers with low functional-group density. The “oil” continues to react internally until it is solidified (arrested). Depending on the speed of solidification and the properties of the medium, the structural diversity that can be generated via this process can be extremely broad. For example, the oily phase may start out as thin, hair-like filaments that evolve into small droplets embedded within fibers; among other possibilities, the latter morphology may evolve into large particles with or without fibers emanating from their surface. By choosing the right monomers and adjusting the reaction rate (e.g., via adjustment of monomer concentration or by using an appropriate catalyst), any skeletal framework with these morphologies can be “frozen” (kinetically trapped) in place before it has time to evolve into the next step. Polyurea aerogels are an extremely versatile class of materials that enable observation and, in some cases, design of those morphologies in the final products. These skeletal morphologies translate directly into a wide range of surface areas, texture-induced hydrophobicity, and diverse mechanical properties that include superelasticity and shape-memory effects. 

### 3.2. Molding, form Factors, and the Drying Process

Nanostructured polyurea aerogels can be produced in various form factors, ranging from monoliths to particles. Shaped monoliths are produced by pouring the polyurea sol into suitable molds and allowing it to gel. Irregular polyurea aerogel particles are produced by stirring the sol vigorously until gelation. Particles in the form of spherical millimeter-size beads may be formed via a dripping method. The use of surfactants for the synthesis of micron-size beads, or of 3D printing—as was reported recently for giving complicated shapes to structurally related polyurethane aerogels [42]—have not yet been reported with polyurea aerogels. 

Drying in most cases has been carried out using SCF CO_2_. Polyurea aerogels have also been produced via freeze-drying by replacing the pore-filling solvent with *tert*-butanol, followed by freezing and subsequent sublimation [43,44]. The freeze-drying process provides easier access to large monolithic panels than supercritical drying; however, the process parameters seem to have a significant influence on the properties of the resulting polyurea aerogels.

## 4. The Literature on Polyurea Aerogels

This section reviews the synthesis, properties, and applications of polyurea aerogels prepared via reaction of isocyanates with amines, water, and mineral acids; it also includes a very brief overview of polyurea-crosslinked aerogels. 

### 4.1. Polyurea Aerogels from Isocyanates and Amines

**Figure 13:**
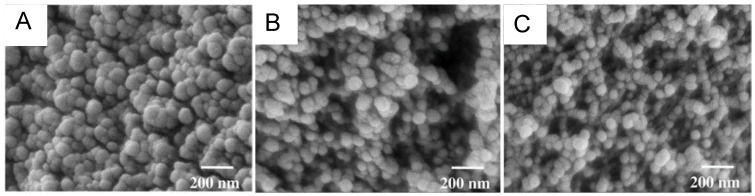
SEM images of (**A**) a silica aerogel at 0.0902 g·cm^−3^, and (**B**,**C**) polyurea aerogels synthesized via reaction of an isocyanate with an amine with constant EW ratio (= equivalent weight of amine/equivalent weight of NCO). The polyurea aerogel in (**B**) was prepared from pMDI and Jeffamine T3000 (aerogel bulk density = 0.06 g·cm^−3^), whereas the aerogel in (**C**) was synthesized from pMDI and Jeffamine T5000 (bulk density = 0.1 g·cm^−3^) [45]. All scale bars represent 200 nm.

Properties of isocyanate-derived aerogels were first reported by Biesmans et al. in the late 1990s [9]; polyurea was referred to in passing without chemical evidence. With an eye on thermal insulation, the first comprehensive study in this subclass of isocyanate-derived aerogels was published in 2009 by Lee et al. of Aspen Aerogels [45]. Lee’s study used two types of isocyanates (4,4′-methylenediphenyl diisocyanate (MDI) and polymeric methane diphenyl diisocyanate (pMDI)) and two types of commercial polyoxopropylene triamines: Jeffamine T3000 and Jeffamine T5000, where the numerical extensions in the product names refer to their approximate molecular weight [46]. Gelation was carried out in acetone with triethylamine as the catalyst. The resulting polyurea aerogels exhibited low shrinkage factors (1.14 ≤ f ≤ 2.95, where f = final density/target density), bulk densities in the range of 0.098–0.116 g·cm^−3^, high porosity (90–91% *v*/*v*), and good hydrophobicity. Polyurea monoliths based on Jeffamine T3000 showed lower shrinkage and lower thermal conductivity values, which was attributed to the higher amount of crosslinked structures. Polyurea prepared from pMDI was more flexible and less dusty than polyurea prepared from MDI itself. Under electron microscopy, these polyurea aerogels looked similar to silica aerogels, consisting of ~50 nm clusters of nano-sized particles; however, high-density polyurea aerogels exhibited larger pores than those of similar-density silica aerogels (Figure 13). 

**Figure 14:**
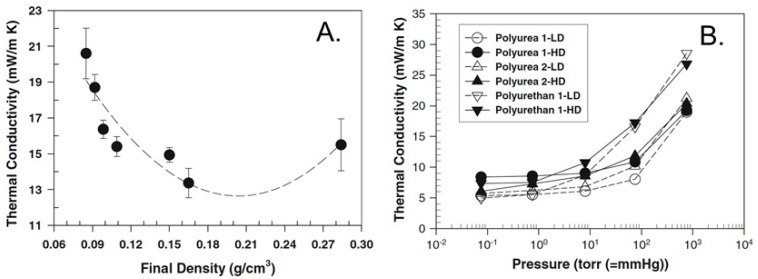
(**A**) Thermal conductivity versus bulk (final) density of various polyurea aerogels prepared with pMDI at a constant EW ratio (= equivalent weight of amine/equivalent weight of NCO) and catalyst (triethylamine) content of 5% *w*/*w*. (**B**) Thermal conductivity of polyurea and analogous polyurethane aerogels prepared with target densities of 0.07 g·cm^−3^ (LD) and 0.1 g·cm^−3^ (HD) as a function of pressure [45].

The thermal conductivity of those MDI/Jeffamine-based polyurea aerogels as a function of bulk (referred to as “final”) density showed a classic U-shaped curve (Figure 14A), typical of aerogels, reflecting a tradeoff between solid thermal conduction, aerogel pore size, and morphology. The thermal conductivity minimum (at 13 mW·m^−1^·K^−1^) was located at around 0.20 g·cm^−3^, which is in the range of other polymeric aerogels, and was comparable to the thermal conductivity of resorcinol–formaldehyde aerogel monoliths (12 mW·m^−1^·K^−1^) and opacified silica aerogels (13 mW·m^−1^·K^−1^) that have been reported previously. The thermal conductivity of various polyurea-based aerogels in this study was compared over a wide pressure range at two different target densities (0.07 and 0.1 g·cm^−3^), along with analogous polyurethane-based aerogels prepared from MDI and a 3400-molecular-weight polyether polyol modified with ethylene oxide (Multranol 9185, supplied by Covestro LLC [47]), using acetone as the solvent. According to Figure 14B, the polyurea aerogels showed lower thermal conductivities than the polyurethane aerogels at all densities and pressures.

More recently, this type of polyurea aerogel was expanded to include polyurea/polyurethane copolymers and isocyanurate-crosslinked polyisocyanate aerogels (the latter were synthesized using trimerization catalysts) [48]. All three types of amine-derived polyurea aerogels were prepared in molds containing non-woven fibrous battings to produce fiber-reinforced polyurea aerogel composites. The resulting materials were evaluated in terms of their thermal conductivity, flexibility, durability, and dustiness for applications in diving gloves and spacesuits. Amongst the three types of aerogels, polyurea aerogels showed the lowest thermal conductivity, but they were also the stiffest materials of the three—both their thermal conductivity and their flexural modulus decreased with increasing target density over the range of 0.04 to 0.08 g·cm^−3^. Increasing the target density beyond this range resulted in decreased flexibility to undesirable levels. A lower isocyanate-to-amine ratio led to improved thermal performance and reduced deformation under impact. Optimization of other formulation variables, including isocyanate functionality, resulted in relatively flexible polyurea aerogels with a thermal conductivity of 18 mW·m^−1^·K^−1^, which remained unchanged after one laundering/drying cycle. The analogous polyurea/polyurethane aerogels (crosslinked with both a polyamine and a polyol) were 10× less dusty than their all-polyurea counterparts, and remained flexible, exhibiting flexural moduli of less than 0.5 MPa at –130 °C, and a bulk density of 0.08 g·cm^−3^. On the downside, the ambient-pressure thermal conductivity of these materials was never lower than 26 mW·m^−1^·K^−1^. 

**Figure 15:**
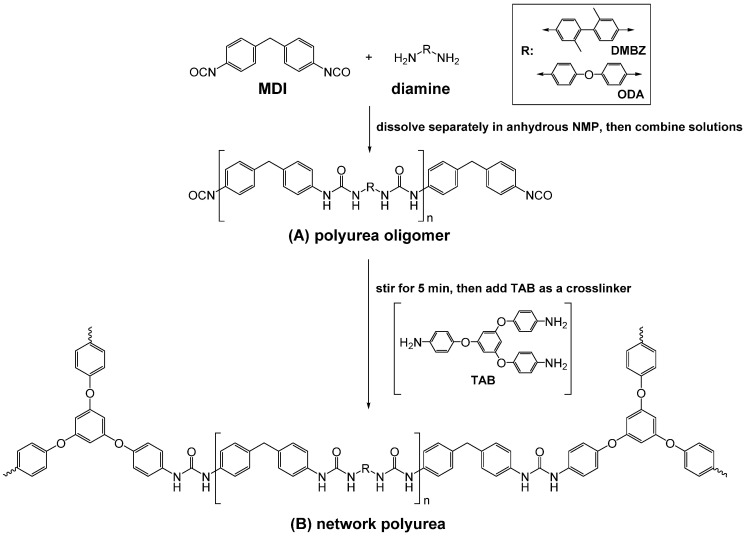
Synthesis of crosslinked polyurea aerogels, showing (**A**) the polyurea oligomer, (TAB) the crosslinker, and (**B**) the network structure [49].

In another study designed to explore the effect of segmental hydrogen bonding (H–bonding) on the properties of polyurea aerogels, gels were prepared in NMP from MDI and one of two different diamines (4,4′-oxydianiline (ODA) and 2,2′-dimethylbenzidine (DMBZ)) [49]. The choice of these two amines was based on the fact that ODA is less bulky than DMBZ, and renders polymeric backbones more flexible thanks to its central oxygen atom. This flexibility should allow for greater segmental motion within the polymer and, therefore, a higher possibility of organization via H-bonding. On the other hand, an advantage of using DMBZ is that it renders polymeric backbones more hydrophobic than those based on ODA. In both cases, the NCO-capped oligomers were crosslinked with 1,3,5-tris(4-aminophenoxy)benzene (TAB), as shown in Figure 15. 

The materials in this study were formulated via the molar ratio of the diamine to MDI, which controls the crosslinking density of the polymer for a constant oligomer-to-TAB ratio of 3:2. The average bulk density of the resulting aerogels was in the range of 0.19–0.26 g·cm^−3^, with porosity in the range of 79–86% *v*/*v*, and surface areas between 106 and 309 m^2^·g^−1^. Microscopically, the ODA-based polyurea aerogels produced in this study consisted of string-of-beads-like, low-aspect-ratio interconnected fibrils, with diameters in the range of 75–125 nm. In contrast, the morphology of DMBZ-based polyurea aerogels varied from fibrillar—with fibril diameters in the range of 10–75 nm—to clusters of particles. Spectroscopic evidence suggested that shrinkage was linked to H-bonding between urea groups, and decreased as H-bonding increased in linear oligomers of increasing molecular weight. As expected, the extent of H-bonding in ODA-based aerogels was higher than in DMBZ-based aerogels, stabilizing the aerogel network and reducing shrinkage compared to the DMBZ-based aerogels (14–21% for ODA-based aerogels versus 21–23% for DMBZ-based aerogels). Accordingly, the density of the DMBZ-based aerogels did not vary with increasing crosslink density. The DMBZ-based aerogels were reported as being somewhat translucent, exhibiting a mean pore size around 15 nm, an onset of thermal decomposition at 250 °C, and a compressive modulus in the range of 38.5–69.4 MPa, compared to 11.7–48.2 MPa for the corresponding ODA-based aerogels.

In an extension of this study, polyurea-*co*-polyurethane block copolymers were developed for improving flexibility [50]. Materials were prepared via a modified version of the procedure shown in Figure 15, as follows: MDI was first reacted with two aliphatic diols (polycaprolactone diol and polytetramethylene glycol, with molecular weights of 530 and 650 g mol^−1^, respectively) to form isocyanate-end-capped oligomers; those oligomers were reacted with ODA, after which the exact procedure of Figure 15 was followed, starting from the top. The densities of the resulting aerogels varied from 0.20 to 0.35 g·cm^−3^, with porosities ranging from 71 to 85% *v*/*v*, and surface areas between 47 and 163 m^2^·g^−1^. The shrinkage of these materials was higher (19–33%) than that of the corresponding all-polyurea aerogels described above (14–21%) [50]. It was reported that shrinkage was correlated with the extent of H-bonding between the urea and urethane groups, leading to the conclusion that the weight fraction of the aliphatic diol can be used as a parameter to control shrinkage. The extent of H-bonding could be also influenced by the degree of crosslinking which, in turn, was affected by the amount of TAB used in the reaction; it was found that reducing the amount of TAB brought about a reduction in urea H-bonding and an increase in urethane H-bonding. The materials were thermally stable up to 300 °C, and exhibited a compressive modulus between 12 and 52 MPa. Microscopically, all formulations comprised networks of branching and interconnected small-aspect-ratio fibrils which, in turn, appeared to consist of assemblies of particles. Samples with a bulk density of 0.22 g·cm^−3^ exhibited a porosity of 82% *v*/*v*, a surface area of 71 m^2^·g^−1^, and a compressive modulus of 10.6 ± 2.0 MPa, and small monoliths with those properties were also reported to be flexible.

**Figure 16:**
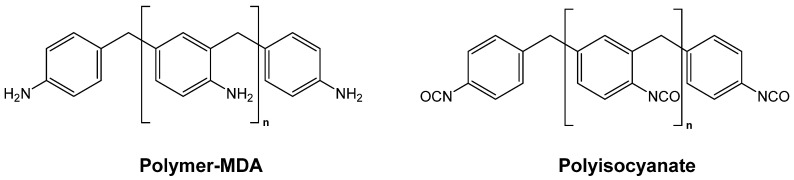
The isocyanate and amine used in the preparation of polyurea aerogels by Wu et al. [51].

A more recent study by Wu et al. targeted improving the thermal conductivity of polyurea aerogels by manipulating their solid framework through controlling gelation time which, in turn, was controlled via the gelation temperature [51]. Polyurea aerogels in this study were prepared from polymethylene polyphenyl isocyanate (PPI) and polymer-MDA (poly-MDA) (Figure 16). The total monomer concentration varied from 5% *w*/*w* to 20% *w*/*w* in DMF (sols with a monomer concentration of 3% *w*/*w* did not gel). Gelation was carried out at ambient temperature, 0 °C, and –20 °C. All aerogels exhibited a fibrous morphology consisting of entangled, worm-like nanostructures. Gelation times increased by 60–80% when decreasing temperature from ambient to 0 °C, and by another 50% when decreasing temperature to −20 °C. Interestingly, shrinkage decreased with decreasing gelation temperature, and for every sol concentration the bulk density decreased correspondingly (all bulk densities were in the range of 0.2–0.08 g·cm^−3^). The thermal conductivity as a function of density showed a typical U-shaped curve for samples synthesized at all three temperatures; the minimum thermal conductivity was reported at 0.0192 ± 0.0002 W·m^−1^·K^−1^, and belonged to samples with a bulk density of 0.096 ± 0.002 g·cm^−3^ prepared at −20 °C using a 10% *w*/*w* concentration of monomers. The particle size of those 10% *w*/*w* samples decreased from ~8 nm to 4 nm to 2 nm when gelled at ambient temperature, 0 °C, and −20 °C, respectively. The particle size/thermal conductivity data of these materials suggest that the latter was controlled by the interparticle contacts along the skeletal framework, consistent with conclusions reached in studies based on nanoparticulate polymer and carbon aerogels [52,53].

**Figure 17:**
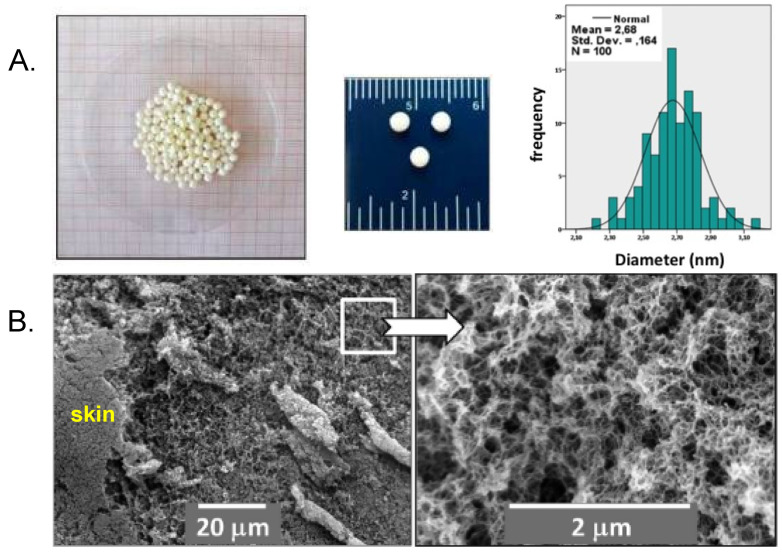
(**A**) Photographs and bead size distribution curve of spherical polyurea aerogel beads prepared via the dripping method. (**B**) SEM images of spherical polyurea aerogel beads showing the denser outer skin of the beads (**left**), and their porous fibrillar interior (**right**) [54].

Finally, in an interesting variation of polyurea aerogel synthesis from isocyanates and amines, a triisocyanate (Desmodur N3300A; see Figure 9) and a diamine (ethylene diamine) were first physically separated in two immiscible liquids—the triisocyanate was dissolved in propylene carbonate, while the diamine was suspended in mineral oil. Then, the isocyanate solution was added dropwise with a disposable pipette (the dripping method) to the diamine suspension in mineral oil, resulting in the formation of spherical beads upon contact of the droplets with the mineral oil phase. As ethylene diamine diffused inside the incoming droplets, the entire volume of the droplets gelled and the droplets were converted to polyurea wet-gel beads. Drying those beads with SCF CO_2_ yielded polyurea aerogel beads with a mean diameter of 2.7 mm and a narrow particle size distribution (full width at half-maximum = 0.4 mm) (Figure 17A) [54]. The bulk density of the beads was 0.166 ± 0.001 g·cm^−3^, with a porosity of 87% *v*/*v* and a BET surface area of 197 m^2^·g^−1^, all within the range of the monolithic isocyanate/amine-derived polyurea aerogels discussed thus far. Microscopically, the internal texture of the beads was fibrillar. The external surface of the beads was denser than the interior (refer to the skin effect [55,56]), and the fibrous morphology was almost completely lost (Figure 17B). 

### 4.2. Polyurea Aerogels from Isocyanates and Water

The mechanistic pathway of this approach to polyurea aerogels is summarized in Figure 8. Polyurea aerogels comprise a large proportion of polymer-based aerogels, and also of composite organic–inorganic aerogels—referred to as polymer-crosslinked aerogels. Mechanically strong polyurea aerogel panels made via this method are commercially available under the tradename Airloy^®^ X103 [57].

The first encounter with this class of aerogels was somewhat accidental, and occurred during efforts to crosslink resorcinol–formaldehyde (RF) aerogels in a fashion analogous to polymer-crosslinked oxide aerogels (e.g., silica). In these experiments, RF wet gels were solvent-exchanged with acetonitrile solutions of Desmodur N3300A (Figure 9) and triethylamine (Et_3_N, catalyst), in an effort to form a conformal polyurea coating over the RF network via reaction of dangling surface –OH groups with the isocyanate. Surprisingly, the crosslinking baths themselves gelled, the cause of which was traced to a small amount of water present in the solvent [19]. Systematic studies of materials based on this chemistry followed shortly thereafter [20,58], resulting in the development of rationally designed polyurea aerogels derived from the reaction of isocyanates with water.

**Figure 18:**
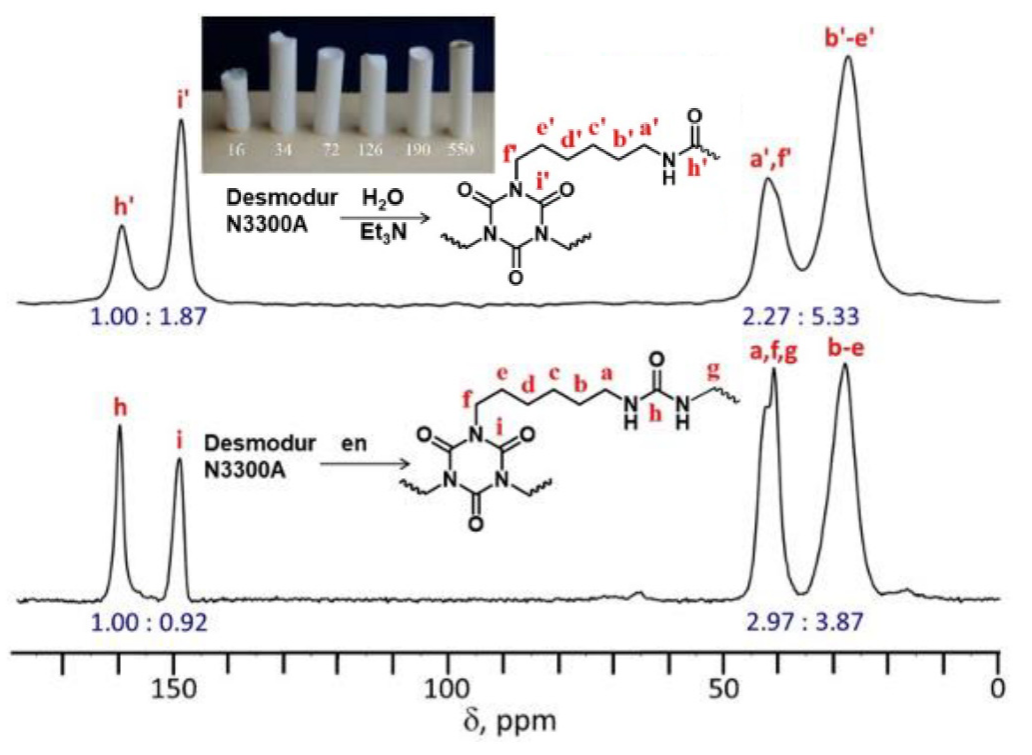
Comparison of the solid-state ^13^C NMR spectra of polyurea aerogels derived from the reaction of the triisocyanate Desmodur N3300A (see Figure 9) with water (top spectrum) and with ethylene diamine (en; bottom spectrum). Relative integrated peak intensities are given underneath each resonance. Inset: photograph of polyurea aerogel monoliths synthesized from Desmodur N3300A and water, spanning the density range of 0.016 g·cm^−3^ to 0.550 g·cm^−3^ [54].

Such Desmodur N3300A/water-derived aerogels are closely chemically related to the polyurea aerogel beads discussed in the previous section (see Figure 17); the difference is that they lack the three –CH_2_– groups per polymer repeat unit coming from ethylene diamine, and include only 1.5 C=O groups per polymer repeat unit instead of 3 (because reaction with water causes loss of CO_2_). These differences are too small to have a significant impact of the properties of the two materials; however, they leave a clear signature in the solid-state ^13^C NMR spectra (Figure 18) [54], underlying the fact that solid-state NMR techniques are instrumental in the chemical characterization of polymeric aerogels.

In the synthesis of Desmodur N3300A/water-derived polyurea aerogels, an approximately stoichiometric amount of water was used (relative to isocyanate) [20]. A large excess of water led to precipitation rather than gelation. Varying the concentration of the isocyanate yielded aerogels with densities ranging from 0.016 g·cm^−3^ to 0.55 g·cm^−3^ (see the photograph used as an inset in Figure 18, top), porosities ranging from 54 to 99% *v*/*v*, and specific surface areas between 54 and 244 m^2^·g^−1^ [20,58]. The variation of surface area with density strongly suggests morphological changes over this density range, as discussed below. 

**Figure 19:**
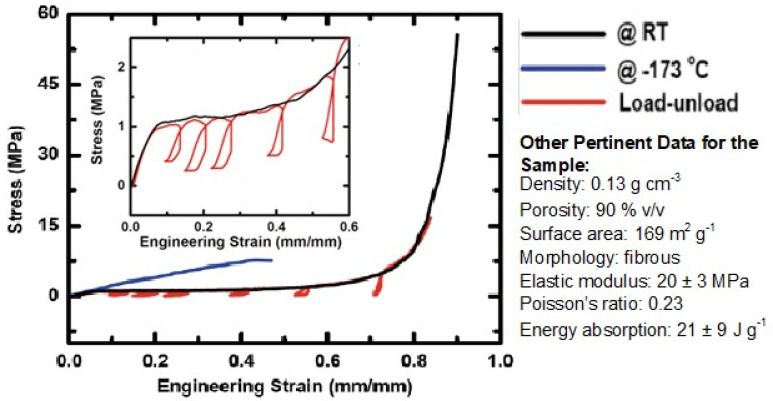
Quasistatic compression testing (strain rate at 0.05 s^−1^) of polyurea aerogel monoliths with bulk density of 0.13 g·cm^−3^, derived from the reaction of the triisocyanate Desmodur N3300A with water in acetone, under various conditions, as color coded. *Inset*: magnification of the elastic region, and of the beginning of the plastic deformation region [20].

Polyurea aerogels produced via the water route were found to be mechanically strong materials. Figure 19 shows a typical compressive stress–strain curve at room and cryogenic temperatures [20]. In similar fashion to polyurea-crosslinked silica and vanadia aerogels [17], at room temperature, Desmodur N3300A/water-derived polyurea aerogels were linearly elastic at small compressive strains (<3%), exhibiting plastic deformation until ~70% compressive strain, followed by densification and inelastic hardening. The compressive Young’s modulus ranged from 4 to 300 MPa following a power-law relationship with density, with an exponent equal to 1.63 ± 0.17, which is lower than that of polyurea-crosslinked oxide aerogels. Poisson’s ratios were small within the linear elastic range (0.20–0.23 up to 5% strain), and remained in roughly the same range as strain increased into the plastic deformation regime (e.g., between 0.16–0.22 up to 70% strain). Negative slopes during unloading (meaning that the material continued to contract despite the load being released) and the ensuing hysteresis loops during repeated load/unload cycles along the stress–strain curve (Figure 19, red line) were attributed to local softening and flow of the polymer for a short period after the stress was released. At cryogenic temperatures (−173 °C), Desmodur N3300A/water-derived polyurea aerogels became stiffer (with Young’s modulus reaching 490 MPa at 0.55 g·cm^−3^) and more brittle, failing by fragmentation at lower strains (42–72%) than at room temperature (88–92%). The overall cryogenic behavior of polyurea aerogels fell between (a) that of polyurea-crosslinked silica aerogels, which become much more brittle at cryogenic temperatures, and fail at just 16% compressive strain (versus 68% at room temperature), and (b) that of polyurea-crosslinked vanadia aerogels, which remain ductile at cryogenic temperatures [17]. Finally, it was noted that the specific energy absorption of higher density N3300A/water-derived polyurea aerogels could be quite high—up to 91 J·g^−1^ at 0.55 g·cm^−3^, which is in fact much higher than that of other materials that are typically considered strong, such as 4130 steel (15 J·g^−1^ at 7.84 g·cm^−3-^), Kevlar-49 epoxy (11 J·g^−1^ at 1.04 g·cm^−3^), and silicon carbide ceramics (20 J·g^−1^ at 3.02 g·cm^−3^, under confinement) [59].

The most intriguing feature of polyurea aerogels derived from Desmodur N3300A and water, which was actually noted from the early investigations into these materials, was that for the given set of synthetic conditions (employing acetone solvent at room temperature), their nanomorphology varied as a function of density from fibrillar at lower densities (below ~0.2 g·cm^−3^) to particulate at higher densities [20]. According to small-angle neutron scattering (SANS) measurements, all samples, irrespective of their nanomorphology, consisted of primary particles assembled into mass fractal spheroidal (or disc-shaped, depending on the concentration of the isocyanate) secondary particles that, in turn, formed either entangled (mass- or surface-fractal) fibrils or higher globular aggregates [20]. 

**Figure 20:**
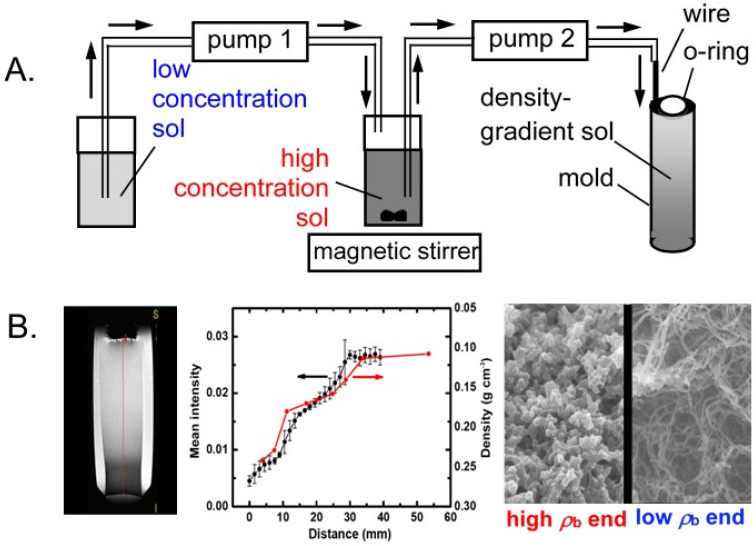
(**A**) Preparation of density gradient polyurea wet-gel monoliths [61]. (**B**) **Left**: magnetic resonance imaging (MRI) of a density gradient polyurea wet gel after it was solvent-exchanged with water (the high-density end of the monolith was at the bottom). **Middle**: density variation by analysis of the MRI images, and by direct measurement (red line: by cutting out and weighing disc-shaped coupons along the length of the monolith). **Right**: SEM images showing the fibrillar morphology at the low-density (*ρ*_b_) end, and the particulate morphology at the high-density end of the monolith [61].

The density dependence of nanomorphology was probed by preparing gradient density cylindrical monoliths of Desmodur N3300A/water-based polyurea aerogels using the method of continuous dilution of the sol (Figure 20A) [60], yielding samples with a linear density gradient imprinted along their length. Consistent with the morphology expected of discrete-density monoliths, the morphology at the high-density end of the density gradient monoliths was particulate, turning to fibrillar as it moved toward the low-density end (Figure 20B) [61]. 

**Figure 21:**
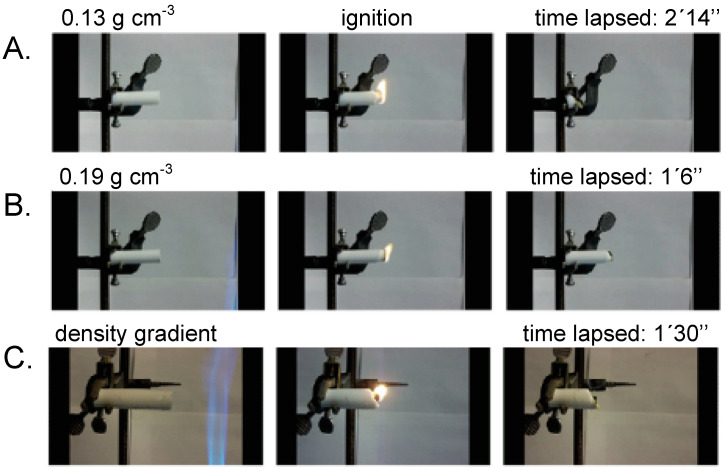
Density-dependent flame propagation of polyurea aerogel monoliths derived from Desmodur N3300A and water. Upon ignition (middle frames), (**A**) samples with bulk densities < 0.2 g·cm^−3^ propagated the flame, and were consumed completely (right frame). (**B**) Samples with bulk densities > 0.2 g·cm^−3^ were self-extinguished shortly after the flame was removed. (**C**) Density gradient aerogels (see Figure 20) burned only until the flame reached the high-density region [61].

An interesting property of the density gradient materials was that when ignited with a flame, (a) lower density samples propagated the flame until they were consumed completely (Figure 21A), (b) higher density samples (>0.2 g·cm^−3^) burned only in direct contact with the flame, and were self-extinguished once the flame was removed (Figure 21B), and (c) density gradient samples ignited from their lower density end propagated the flame, and burned only until the flame reached a region with sufficiently higher density, where it was self-extinguished (Figure 21C) [61]. It was speculated that the origin of this density-dependent flame retardancy was related to the nanomorphology dependence of the air circulation through the porous structure.

Subsequently, the simple synthetic protocol of polyurea aerogels via the water route—together with their good mechanical properties, flame retardancy, and intriguing nanomorphology—became the point of departure for numerous other studies on their processing, structure–property relationships, and applications. 

Most commonly, polyurea aerogels are prepared in monolithic forms. Higher density monolithic samples (>0.1 g·cm^−3^) can be machined into desirable shapes using regular tools. Non-contact slicing of lower density polyurea aerogel samples has been performed at ambient conditions using an 800 nm Ti:sapphire femtosecond laser [62]. The ablation rate was investigated at different energy levels, and was found to be in the order of tens of microns per 40 fs pulse. Periodic grooves noted on the newly exposed cut surfaces were attributed to a material removal mechanism that involves melting and vaporization.

**Figure 22:**
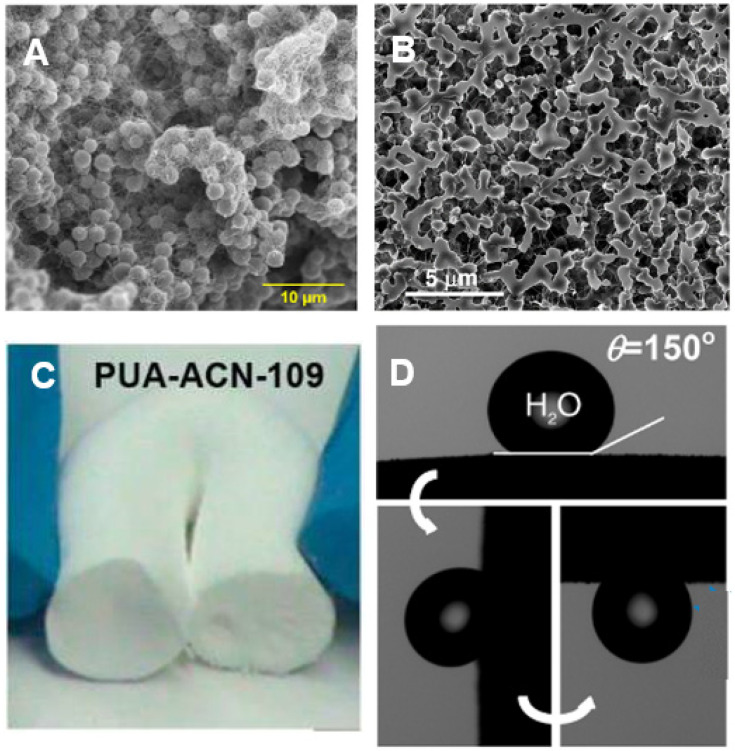
(**A**) SEM of a Desmodur N3300A/water-derived polyurea aerogel (bulk density = 0.172 g·cm^−3^) prepared in acetonitrile. (**B**) SEM of a similar sample as in (**A**) after cross-sectioning with an Ar-ion beam, revealing that the aerogel’s constituent particles were dense, with no internal structure. (**C**) Low-density (0.073 g·cm^−3^) polyurea aerogel monoliths synthesized in acetonitrile were flexible. (**D**) Contact angle of a water droplet on the acetonitrile-synthesized polyurea aerogel monolith from frames (**A**) and (**B**). The water droplet remained attached to the surface even when the sample was flipped upside-down (the rose petal effect) [63].

The fact that the morphology of this class of polyurea aerogels changes with density became even more intriguing when it was observed that the nanostructure can also be varied by simply changing the gelation solvent holding all other parameters constant. Specifically, it was noted that Desmodur N3300A/water-derived polyurea aerogels synthesized in acetonitrile could take on a cocoon-like nanostructure (Figure 22A), wherein a solid core (Figure 22B) is trapped inside a fibrous web [63]. These acetonitrile-synthesized polyurea aerogels were flexible (Figure 22C), superhydrophobic (water contact angle of 150°), and demonstrated the rose petal effect (Figure 22D). 

The diverse morphologies accessible by Desmodur N3300A/water-based polyurea aerogels enable the study of structure–property relationships in nanostructured matter, while holding chemical composition constant. In this context, the Reichenauer group at ZAE Bayern investigated the thermal conductivity of Desmodur N3300A/water-derived polyurea aerogels as a function of nanomorphology [64]. Thermal conductivity values between 0.027 and 0.066 W·m^−1^·K^−1^ were obtained using the hot-wire method for samples of densities between 0.04 and 0.53 g·cm^−3^. The total thermal conductivity was deconvolved into gaseous, radiative, and solid conductive transport contributions as a function of pressure and temperature. The solid thermal conductivity scaled with density with an exponent α = 1, which is significantly lower than the typical exponents found for aerogels at around α = 1.5 [53,65,66]). It was concluded that unlike macroporous foams, which exhibit linearly increasing solid-phase thermal conductivity with increasing density because of their regular pore structure [67], the transition of microstructure in N3300A/water-derived polyurea aerogels from fibrillar to particulate with increasing density counteracts this trend. Specifically, the particulate nanomorphology in higher density samples results in numerous interparticle contact points, which serve as effective heat resistors in a network model of thermal conduction. These added contact points work to partially counteract the increase in solid-phase thermal conductivity that would otherwise result from increased density. Thus, the reason the exponent α = 1 for N3300A/water-derived polyurea aerogels is not related to the reason that α = 1 for foams.

Subsequently, the same group investigated the relationship between thermal conductivity and compressive stiffness as a function of density, following two different approaches: In the first approach [68], polyurea aerogels with an initial density of 0.027 g·cm^−3^ were compressed uniaxially to various strains up to a maximum density of 0.400 g·cm^−3^. The properties of interest in this study included microstructure, thermal conductivity, and elastic modulus. The important finding in this study was that thermal pathways along the skeletal backbone of the aerogels can be decoupled from the mechanism that supports external compressive loads (note that similar conclusions had been reported previously with silica aerogels [69]). Thus, at low densities, the stiffness of the compressed polyurea aerogels and their solid thermal conductivity followed a quadratic relationship with one another, which was attributed to the fact that microscopic deformations were dominated by bending, which does not alter the length of thermal conduits in the material. At higher densities (achieved by higher compressive strains in the context of these experiments), the relationship between stiffness and solid thermal conductivity transitioned from quadratic to linear, which was attributed to network compaction. The key parameters that allowed for a microstructural decoupling of mechanical and thermal properties were identified as the microscopic homogeneity of the solid network and the curvature of its network elements. In the second approach [70], similar studies were conducted with two series of samples prepared in the density range of 0.03 to 0.3 g·cm^−3^. Again, the elastic modulus was found to correlate with solid thermal conductivity, irrespective of microstructural details. It was speculated that possibilities for structural decoupling of the elastic modulus and heat transport through the solid phase, as suggested by these studies, could open new approaches to applications, presumably in the spirit of reference [69]. 

**Figure 23:**
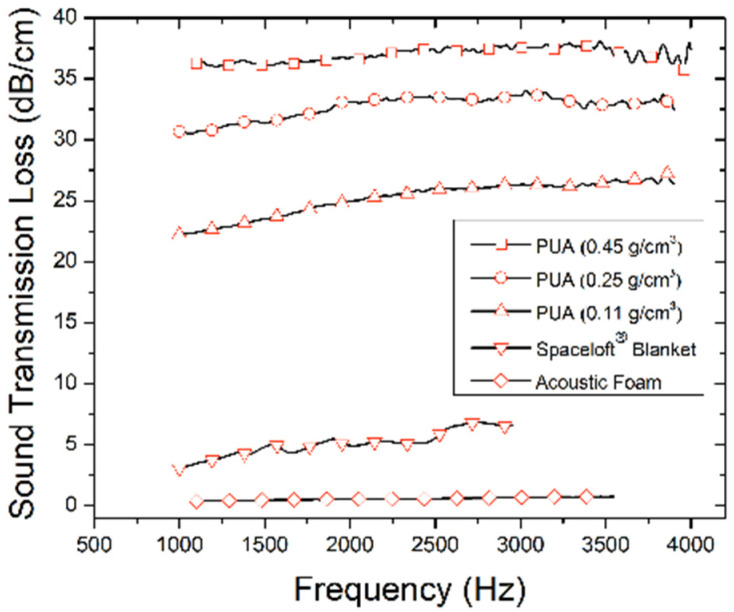
Experimental sound transmission loss data of Desmodur N3300A/water-derived polyurea (PUA) aerogels at the densities indicated in the legend, compared with two other relevant commercial porous materials (Spaceloft^®^ blanket and acoustic foam, Northborough, MA, USA) [71].

The mechanical properties of N3300A/water-derived polyurea aerogels have also been investigated from the perspective of acoustic attenuation and shockwave absorption. Lu et al. have reported sound transmission loss values of over 30 dB·cm^−1^ over the range of 1 to 4 kHz for such polyurea aerogels with a bulk density of 0.25 g·cm^−3^ and a thickness of 5 mm, which is considered extremely high for such a material (see Figure 23 for a comparison with other relevant materials) [71,72]. Inspired by the nanostructure of Figure 22A, and noting striking similarities with acoustic metamaterials, polyurea aerogels were modeled first with a one-dimensional multi-degree-of-freedom mass-spring system. Wave transmission loss results were produced for different configurations modeled to reflect various polyurea aerogel nano- and microstructures. Significant wave attenuation was observed with a random spring distribution. Based on these results, the authors introduced polyurea aerogels of different bulk densities and porosities into laminated composites in the form of thin sheets—for example, placed between two gypsum wallboards of the type typically used in soundproofing applications [73]. The sound transmission loss of the sandwich structure reached 40 dB·cm^−1^ at 2 kHz with just two 5 mm thick aerogel layers with bulk densities of 0.15 and 0.25 g·cm^−3^, respectively. In parallel, an exact analytical time-harmonic plane-strain solution for the diffused wave propagation through the multilayered structure was developed using the theories of linear elasticity and Biot’s dynamic poroelasticity. The theoretical results were well supported by experiments, and the authors suggested that such materials could be utilized for the design of future lightweight multifunctional composite structures [73].

In a similar study incorporating both experiment and theory, commercially available Desmodur N3300A/water-based polyurea aerogels (Aerogel Technologies Airloy X103 [57]) were considered for use in explosive shockwave mitigation, and were tested using single- and two-stage gas-gun-driven plane impact experiments [74,75,76,77]. In response to the intense interest in the mechanical properties of the Desmodur N3300A/water-based polyurea aerogels, more recently, the nonlinear mechanical properties, deformation mechanisms, and failure modes of such polyurea aerogels were investigated in detail using a multiscale approach that combined experimental nanoindentation, analytical modeling, and computational modeling [78]. First, primary particles were modeled numerically from the monomer upwards, and their mechanical interactions were investigated with molecular dynamics simulations. From nanoindentation, four deformation and failure modes were identified: free-ligament buckling, cell-ligament bending, stable-cell collapse, and ligament-crushing-induced strain hardening. The corresponding structural evolution during indentation and strain hardening were analyzed and modeled. The material scaling properties were found to be dependent on both the bulk density and the secondary particle size. The best fit of the data was achieved with secondary particles comprising 10 primary particles. Using a porosity-dependent material constitutive model, a linear relationship was found between the strain hardening index and the secondary particle size, rather than a conventional power-law relationship. Finally, the structural efficiency of Desmodur N3300A/water-based polyurea aerogels with respect to their energy absorption capability was evaluated as a function of structural parameters and base polymeric material properties.

Because of their high mechanical strength, Desmodur N3300A/water-based polyurea xerogels and aerogels have been considered for use in concrete confinement [79]. Later, inspired by the structure of nacres, this literal bricks-and-mortar concept of composite design employing Desmodur-N3300A/water-based polyurea aerogels was translated into nanocellular composites by infiltrating preformed polyurea networks in acetonitrile with a hard inorganic phase, namely, magnesium phosphate cement particles. In essence, the material design here was a conceptual inverse to polymer-crosslinked oxide aerogels. Owing to nanoconfinement effects, the effective compressive modulus and compressive strength of the polyurea ligaments (measured using nanoindentation) were found to be 8–10 times higher than those in the non-infiltrated aerogels, while both the low density (0.285 g·cm^−3^) and the high porosity (86.4% *v*/*v*) of the parent aerogel were preserved. Overall, this bioinspired nanocomposite design exhibited synergistic properties, offering both high strength and deformability. This nanoconfinement effect was subsequently modeled analytically [80].

With an eye towards biomedical applications, Yin and Rubenstein evaluated the biocompatibility of Desmodur N3300A/water-derived polyurea aerogels as a function of their density and morphology in the range of 0.035–0.21 g·cm^−3^, in the form of their effects on the vascular system, by looking at their hemolysis, platelet activity, endothelial cell activity, and inflammatory responses [81]. Desmodur N3300A/water-derived polyurea aerogels did not absorb proteins, alter blood cells, or cause inflammatory responses. All polyurea aerogels were compatible with endothelial cells, although changes were observed in the gel morphology after contact with human platelet-poor plasma for 48 h, followed by washing with deionized water and drying in air. It was not clear whether those morphological changes, akin to structural collapse during xerogelling, were brought about by the contact with the plasma or by the post-processing method and drying. Overall, it was concluded that Desmodur N3300A/water-derived polyurea aerogels are suitable for cardiovascular applications.

**Figure 24:**
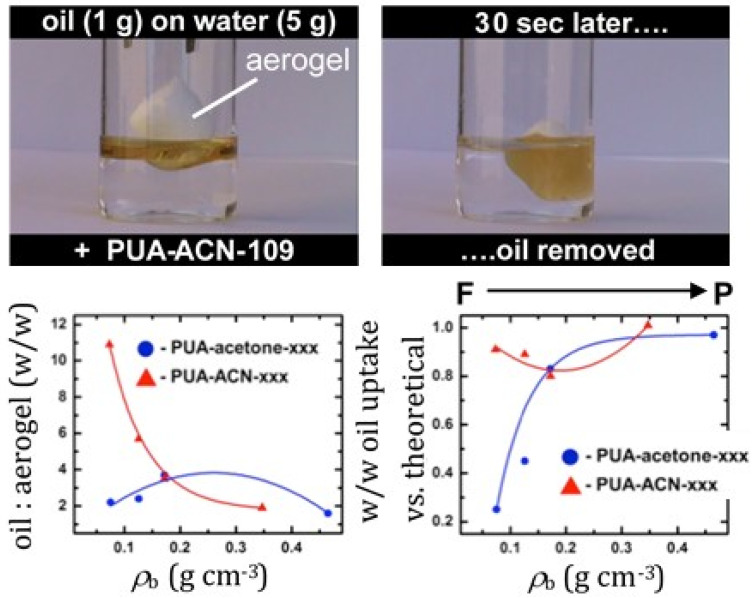
**Top**: removal of pump oil from water with a chunk of polyurea (PUA) aerogel made in acetonitrile (ACN; numerical extensions in sample names are related to the sol concentration). The aerogel sample in this figure had a weight of 0.087 g, a volume of 1.19 cm^−3^, a density of 0.073 g·cm^−3^, a porosity of 94% *v*/*v*, and exhibited an uptaken oil-to-aerogel weight ratio of 11.5 *w*/*w* (see Figure 22A for an SEM image representative of the nanomorphology of this material). **Bottom**: **Left**: gravimetric oil absorption as a function of bulk density. **Right**: ratio of experimental versus theoretical oil uptake (the latter was calculated from the porosities of the samples and the density of the oil, which was 0.924 g·cm^−3^). The arrow above the right frame shows the direction of the morphological transition from fibrillar (F) to particulate (P). The labels PUA-acetone and PUA-ACN refer to polyurea aerogels made in acetone and acetonitrile, respectively [63].

Based on the superhydrophobicity of certain polyurea aerogels of this class (see Figure 22D above), samples with the cocoon-in-web morphology (made in acetonitrile, [63]) were tested for their oil absorption capacity, and results were compared with those from samples made in acetone that exhibited the typical morphological variability from fibrous to particulate, as discussed throughout this section (see Figure 20 [20,61]). Low-density cocoon-in-web samples (0.073 g·cm^−3^) were found to uptake 11 times their weight in pump oil (Figure 24), competing favorably with other aerogel materials evaluated for such applications—especially in terms of volumetric capacity [63]. As shown in Figure 24, both density and morphology again played a significant role in oil uptake, with the ultimate oil capacity found to be related to overall porosity.

**Figure 25:**
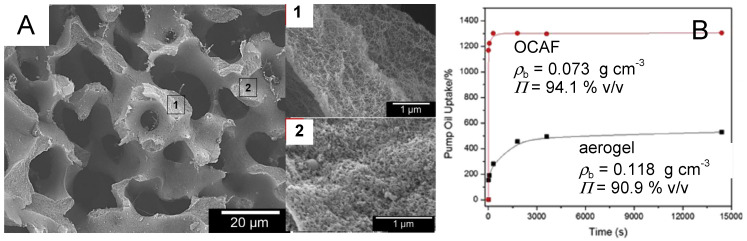
(**A**) SEM image of a polyurea OCAF (*ρ*_b_ = 0.0073 ± 0.003 g·cm^−3^, porosity *Π* = 94.1% *v*/*v*). *Inset 1*: fractured surface of an aerogel wall domain. *Inset 2*: denser skin layer of the aerogel domain. (**B**) Weight percentage of pump oil absorbed as a function of time by OCAFs and corresponding non-templated Desmodur N3300A/water-derived aerogels at the densities and porosities shown within the frame [82].

More recently, the Jana group at the University of Akron revisited the subject of oil uptake from Desmodur N3300A/water-based polyurea aerogels, with an innovative twist—they developed a new class of hierarchically porous composite materials referred to as open-cell aerogel foams (OCAFs), which combine the attributes of open-cell polymer foams (pore size > 1 μm) with those of mesoporous Desmodur N3300A/water-based polyurea aerogels (pore size ~50 nm) [82]. OCAFs were prepared using templating with co-continuous immiscible polymer blends. The open-cell macropores were created by selective dissolution of polystyrene from a co-continuous blend of polyethylene oxide with tetrahydrofuran. The polyurea network was synthesized inside the macropores from an acetone sol. The polyethylene oxide phase was then dissolved away with water, and the resultant material was dried with SCF CO_2_ to obtain polyurea OCAFs. The microstructure of a typical polyurea OCAF is shown in Figure 25A. Internally, the walls that define the macroporous network consisted of typical mesoporous Desmodur N3300A/water-derived polyurea fibers exhibiting the material properties expected for the given densities. The surface of these walls clearly showed a skin effect, comprising denser polymer at the outer geometric boundary (compare with Figure 17). As shown in Figure 25B, OCAF samples with a bulk density of 0.073 g·cm^−3^ showed a 14× *w*/*w* of oil uptake versus the 11× *w*/*w* uptake displayed by cocoon samples of the same bulk density (see Figure 24). This difference in uptake performance can be attributed in part to the extra void space available in the OCAFs. Notably, the corresponding non-templated polyurea aerogels showed a much lower oil uptake, in the same range as the study by Leventis et al. (see Figure 24, lower left frame [63]), highlighting the reproducibility of these materials.

**Figure 26:**
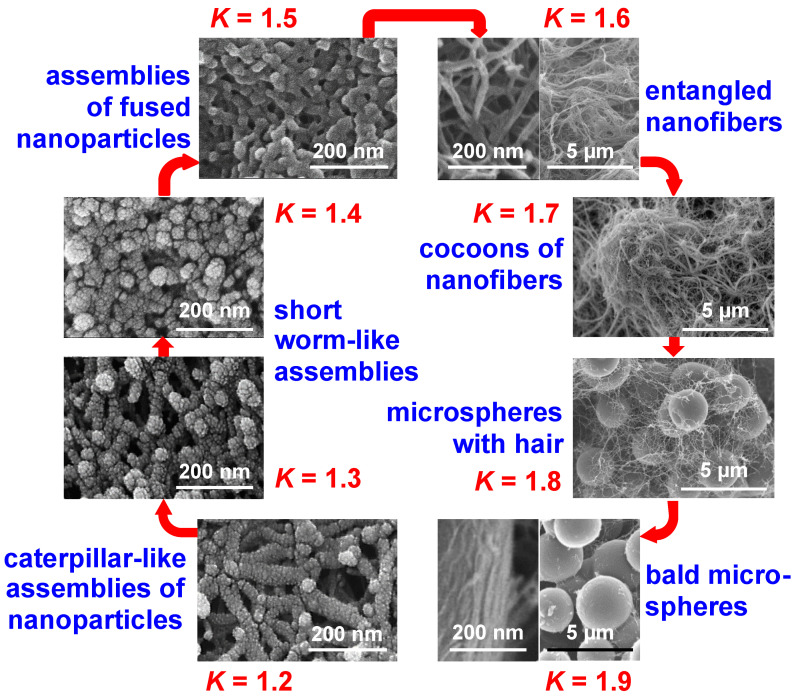
The 8 nanomorphology groups identified from 188 formulations of Desmodur N3300A/water-derived polyurea aerogels prepared using 8 different solvents (i.e., acetone, acetonitrile, nitromethane, propylene carbonate, THF, DMF, 2-butanone, and ethyl acetate). *K*-index is defined as the ratio of the water contact angle to the percentage of porosity for a given material (*θ*/*Π*). Each morphological group shown in this figure was associated with a unique *K*-index value [83].

Finally, in response to the broad interest in the structure and properties of Desmodur N3300A/water-based polyurea aerogels, the Leventis group sought to find ways to express structure–property relationships quantitatively; this pursuit led to the problem of how to prepare samples with given nanostructures at will. In this context, it was realized that in order to establish procedures that deterministically relate nanomorphology to synthetic conditions, it would be necessary to express nanostructure numerically. Guided by a statistical design-of-experiments (DoE) model, a large array of Desmodur N3300A/water-based polyurea aerogels was prepared (188 samples initially), in which the solvent, the concentrations of the monomers in solution, and the catalyst were varied systematically [83]. At first, the structural variability seemed overwhelming; however, upon reflection on the SEM images of those samples, it was realized that one’s first impression about a nanostructure is related to its openness and texture—the former is quantifiable by the porosity (*Π*), while the latter is often related to hydrophobicity which, in turn, can be quantified by the contact angle (*θ*) of water droplets resting on the material (see Figure 22D). The simple numerical ratio of *θ-*to*-Π*, referred to as the *K-index* (“*K*” being phonetic for the first letter of the word “correlator”), could then be used to place all 188 polyurea aerogels of the DoE model into 8 *K*-index groups associated with nanomorphologies ranging from caterpillar-like assemblies of nanoparticles, to random assemblies of fused nanoparticles, entangled thin nanofibers, microspheres with hair, cocoon-like structures (such as the one shown in Figure 22A), and large bald microspheres (see Figure 26). 

**Figure 27:**
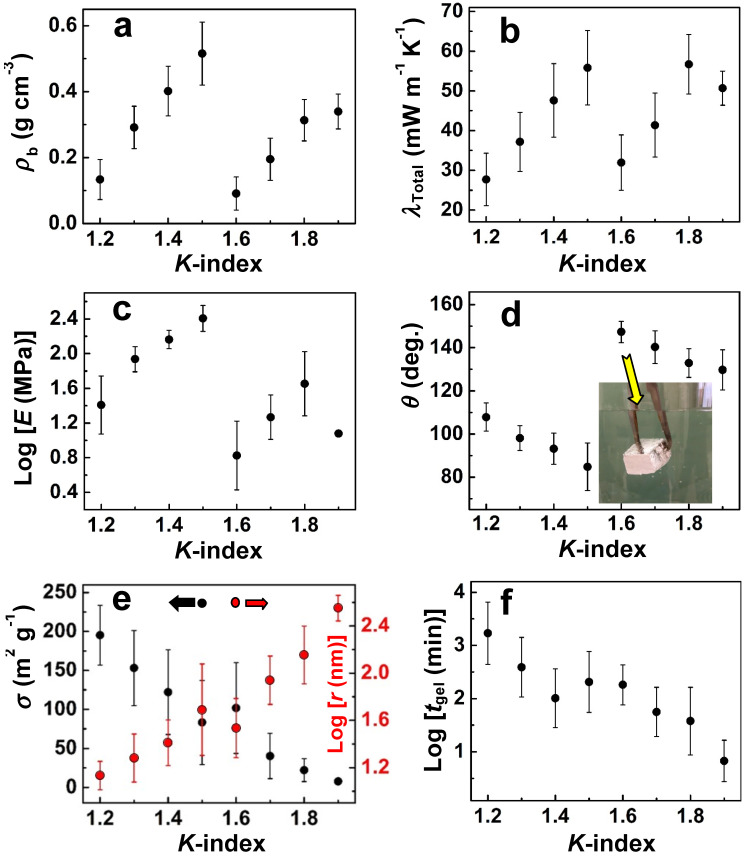
Selected material properties for all 208 Desmodur N3300A/water-derived polyurea aerogel samples prepared using single and binary solvent systems, as a function of their *K*-indices [83]. (**a**) Bulk density, *ρ*_b_. (**b**) Total thermal conductivity, *λ*_Total_. (**c**) Compressive Young’s modulus, *E*. (**d**) Contact angle, *θ*, of a water droplet on an exposed internal surface of a monolith of the given material. *Inset*: total reflection from the layer of air trapped on the surface of a hydrophobic aerogel block submerged in water. The yellow arrow shows which group of *K*-index values the sample corresponds to. (**e**) BET surface area, *σ*, and particle radius, *r*. (**f**) Phenomenological gelation time, *t*_gel_ (*K*-index values were pinned to single-digit decimals in order to facilitate discussion).

At first, the *K*-index was validated as a morphological descriptor by compressing samples to different strains and observing that as the porosity decreased, the water contact angle decreased proportionally and, thus, the *K*-index remained constant, as expected based on the fact that morphology should not change by compression—at least not in the early stages [83]. The predictive power of the *K*-index was demonstrated by preparing 20 randomly formulated Desmodur N3300A/water-based polyurea aerogels in 8 randomly selected binary solvent systems; the *K*-indices of the resulting 20 randomized samples were then determined experimentally (by calculating *θ*/*Π*). The expected structures based on the *K*-index values (as shown in Figure 26) were then compared with SEM images of the samples, and were found to match. At this point, these additional 20 samples that were prepared in binary solvents were added to the pool of 188 samples studied initially, thus increasing the selection of samples from which to extract material properties to 208 samples in total. Next, several material properties of interest were correlated with nanomorphology using the *K*-index (see Figure 27). Eventually, the structural diversity of this type of polyurea aerogel was related to the phase separation mechanism involved in gel formation (see Figure 12). It should be noted that numerous attempts in the literature to infer nanomorphology from quantifiable material properties have focused on the mechanical properties, which have therefore been assumed to be the link between nanomorphology and synthetic conditions [84,85]. However, according to Figure 27, most material properties of interest of polyurea aerogels are not single-value functions of the *K*-index (i.e., of the nanomorphology). Therefore, because in at least one case (polyurea) the mechanical properties cannot be used as uniquely defined descriptors of nanomorphology, they cannot be assumed a priori to be such descriptors in any other case.

Next, all material properties of practical interest in Figure 27 were fitted to the six independent variables of the system using quadratic models: the concentrations of Desmodur N3300A, water, and catalyst used in each sol, along with the three Hansen solubility parameters (HSPs) of the sol (that is, the three parameters calculated for the specific mixture comprising solvent, Desmodur N3300A, water, and triethylamine) [83]. Correlating aerogel properties (bulk density and thermal conductivity) with the properties of the solvent via the HSPs was first reported with PIR-PUR aerogels prepared using a sub-stoichiometric amount of polyol; however, with no quantitative tools available, only qualitative correlations could be made between the properties of interest and the aerogel’s nanomorphology [86]. On the other hand, in reference [83], the resulting six equations (one for each property) enabled the synthesis of polyurea aerogels with up to six prescribed material properties at a time, including nanomorphology, bulk density, BET surface area, compressive modulus, ultimate compressive strength, and thermal conductivity. If a solution of the system of the six equations with the six unknowns included a complex number or a negative value, materials with that particular combination of properties could not be made. This study helped identify the lowest thermal conductivity value of Desmodur N3300A/water-based polyurea aerogels within the domain of this study as 18.5 W·m^−1^·K^−1^, for a material exhibiting a caterpillar-like fibrous morphology (*K*-index = 1.22) and a low bulk density (0.082 g·cm^−3^) synthesized in THF [83]. 

The formation of polyurea aerogels via reaction of triisocyanates with water has been applied successfully with other triisocyanates as well, including Desmodur N3200 (see Figure 7), Desmodur RE (Figure 9), and toluene diisocyanate [20]. Using the fully aromatic Desmodur RE triisocyanate, it was possible to prepare aromatic polyurea aerogels over the entire density range obtained with Desmodur N3300A (from about 0.02 g·cm^−3^ to over 0.25 g·cm^−3^). Using either Desmodur N3200 or toluene diisocyanate, only aerogels with bulk densities of ≥0.2 g·cm^−3^ could be made. Polyurea aerogels from Desmodur RE are particularly interesting, because they can be pyrolyzed to carbon aerogels, and are therefore reviewed in more detail below.

**Figure 28:**
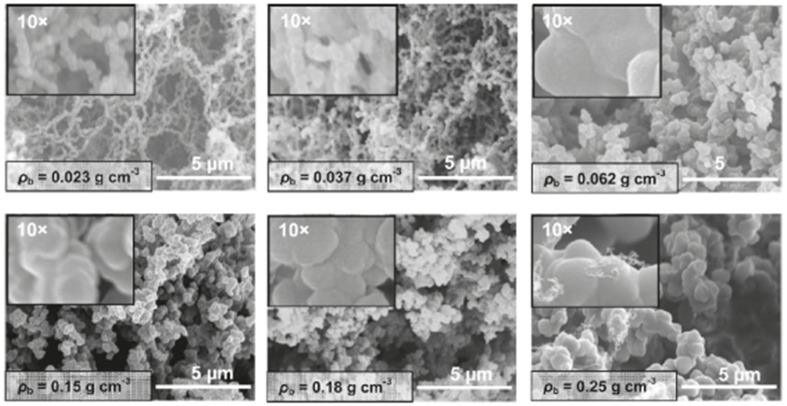
SEM images of polyurea aerogels prepared from Desmodur RE using 3.0 mol equivalents of H_2_O and 0.6% (*w*/*w*) Et_3_N in acetone [20], ordered by increasing bulk density. All scale bars are 5 microns.

For the purposes of cross-referencing with the next section, polyurea aerogels derived from the reaction of Desmodur RE with water are referred to as PUA-yy, and their properties are used again in Section 4.3 as a benchmark for the properties of polyurea aerogels derived from the reaction of Desmodur RE with mineral acids (referred to as BPUA-xx). At low densities, PUA-yy exhibited a fibrillar morphology, progressively turning particulate as the density increased (Figure 28). All other parameters remained constant, and the transition from fibrillar to particulate morphology occurred at lower densities than in Desmodur N3300A/water-derived polyurea aerogels—by 0.062 g·cm^−3^, Desmodur RE/water-derived materials were particulate. The Desmodur RE-derived fibrils consisted of well-defined strings of nanobeads connected by narrow necks (refer, for example, to the 0.023 g·cm^−3^ sample in Figure 28). As the density increased to 0.037 g·cm^−3^, the bead size remained roughly the same, but the interparticle neck zones became wider. Upon further increase in density, Desmodur RE/water-derived polyurea aerogels appeared nanoparticulate, exhibiting only a very faint remanence of the string-of-beads nanostructuring, if any.

**Figure 29:**
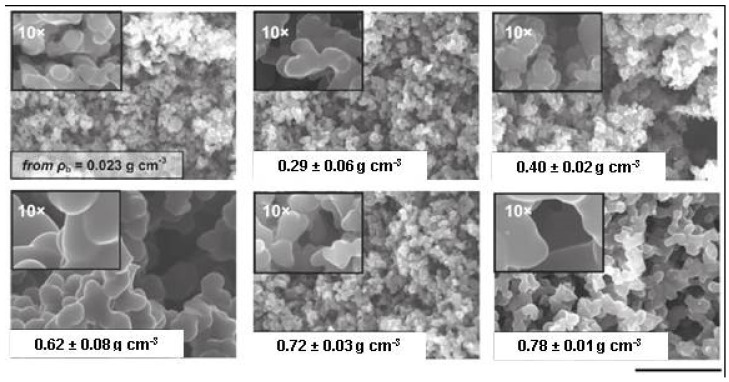
SEM images of carbon aerogels derived from polyurea aerogels via reaction of Desmodur RE and H_2_O in acetone. The common scale bar for all images in the lower right-hand corner represents 5 μm. These SEM images correspond frame-by-frame to those of their parent polyurea aerogels shown in Figure 28 [20]. Note that the carbon aerogel derived from the polyurea aerogel with a density of 0.023 g·cm^−3^ broke into pieces during processing and, therefore, the density of its carbonized derivative was not measured.

Generally, the common feature of carbonizable polymers is that they can undergo either heat-induced cyclization or ring fusion and subsequent chain coalescence [87]. For these processes to take place, the polymer chain should either contain aromatic moieties linked by just one carbon atom, or be aromatizable (usually oxidatively, e.g., as in the case of polyacrylonitrile, polybenzoxazines, and certain phenolic resins). Desmodur RE-derived polyurea aerogels fall into the former category; their pyrolytic yield (at 800 °C under Ar) was found to be around 56% *w*/*w* [20]. The resulting carbon aerogels consisted of a mixture of C (78–82% *w*/*w*), N (5–9% *w*/*w*), and O (~5–9% *w*/*w*), and contained no detectable H. The atomic ratio of O to N was around one-to-one, as determined by XPS. XRD showed only very broad diffractions. Raman spectroscopy showed both the G-band (graphitic) and D-band (disordered) peaks at 1597 cm^−1^ and 1352 cm^−1^, respectively, with an integrated peak intensity ratio I_D_/I_G_ of 1.12. Together, these data suggest nanocrystalline/amorphous carbon, supported by skeletal density measurements (1.78–1.89 g·cm^−3^) that were found to be consistent with amorphous carbon (1.8–2.0 g·cm^−3^) [88]. The bulk densities of these carbon aerogels were higher (in the range of 0.3–0.8 g·cm^−3^) than the densities of their parent polyurea aerogel precursors (in the range of 0.02–0.25 g·cm^−3^). By SEM (Figure 29), all Desmodur RE/water-derived carbon aerogels appeared to be macroporous materials, and were all very similar in appearance, irrespective of their parent polyurea aerogel. In particular, the two lowest density samples completely lost the string-of-beads structure exhibited by their parent polyurea aerogels, although they did retain a faint memory of the wider necks observed, for example, in the 0.037 g·cm^−3^ samples (compare Figure 28 and Figure 29). The morphology of the nanoparticulate structures in samples with densities above 0.062 g·cm^−3^ (refer to Figure 28) was retained more closely. It was proposed that the morphology equalizer across densities was the sintering processes during pyrolysis. 

### 4.3. Polyurea Aerogels from Isocyanates and Mineral Acids

**Figure 30:**
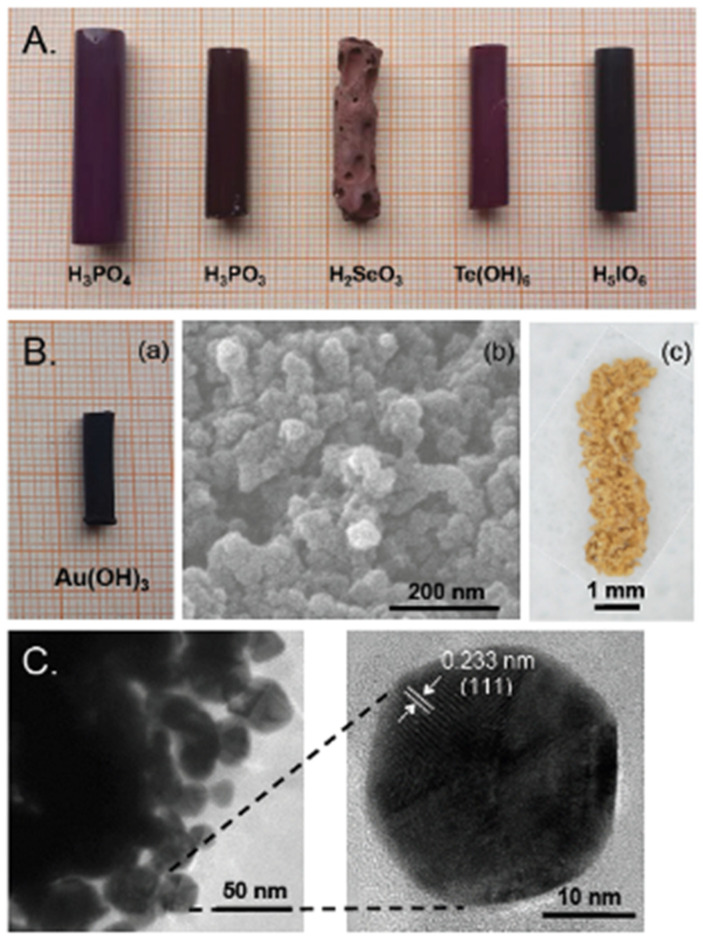
(**A**) Polyurea aerogel monoliths prepared in DMF from Desmodur RE (Tris(4-isocyanatophenyl)methane, abbreviated as TIPM, see Figure 9 and Figure 10) and the acids listed underneath the samples. (**B**) A polyurea aerogel monolith prepared in DMF from TIPM and H_3_AuO_3_ (**a**), its microstructure (**b**), and the residue obtained after pyrolysis at 600 °C in air, consisting of pure gold (**c**). Although the material underwent partial sintering and significant shrinkage, panel B(**c**) proves that Au was evenly distributed throughout the monolith. (**C**) TEM of Au clusters in the Au-doped polyurea aerogel shown in panel B(**a**). The d-spacing of the clusters was found to be close to the literature value (0.2355 nm) for the (111) face of fcc Au(0) [30].

As discussed in Section 2.4, multifunctional isocyanates react with anhydrous mineral acids (e.g., H_3_BO_3_, H_3_PO_4_, H_3_PO_3_, H_2_SeO_3_, H_6_TeO_6_, H_5_IO_6_, and H_3_AuO_3_) to yield urea wet gels, typically doped with the corresponding oxide (i.e., the anhydride) of the mineral acid in use. Exceptions were boric acid, wherein the oxide was washed out during solvent exchange, and auric acid, wherein the resulting dopant was metallic gold (Figure 30) [30,89].

**Figure 31:**
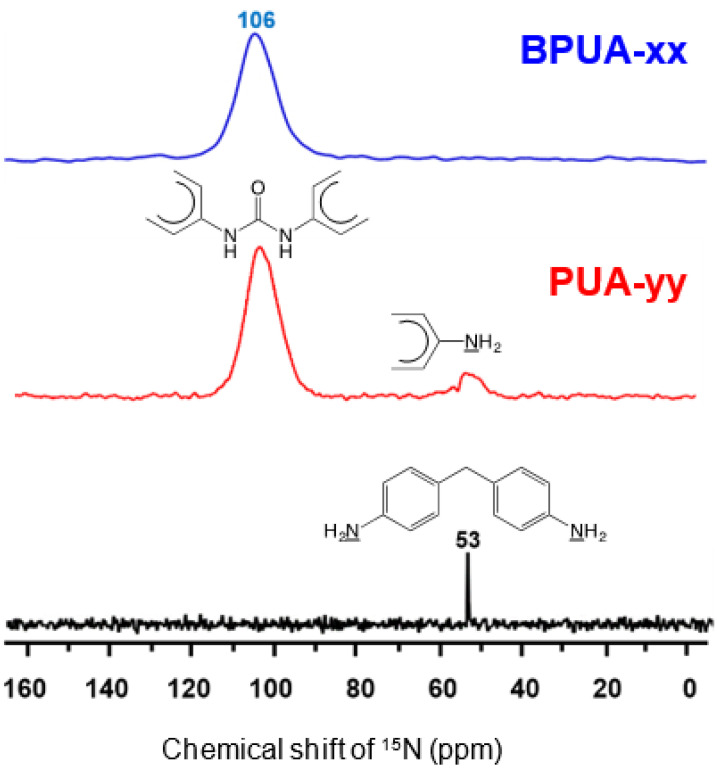
Solid-state CPMAS ^15^N NMR spectra of boric-acid-derived BPUA-xx (top spectrum) and water-derived PUA-yy (middle spectrum), along with the liquid-phase ^15^N NMR spectrum of methylenedianiline in CD_3_NO_2_ (bottom spectrum), used as a control for identifying dangling aromatic amines. Chemical shifts are reported versus liquid ammonia [30] (see text for the samples represented by the abbreviations).

**Figure 32:**
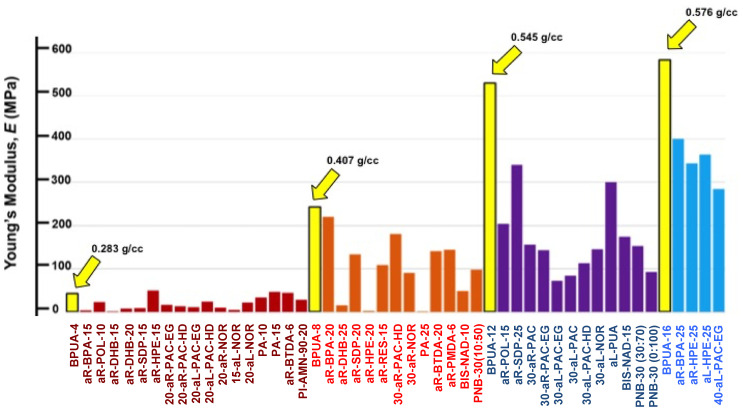
Young’s moduli for four density groups (-xx) of polyurea aerogels obtained from the reaction of Desmodur RE (TIPM) with boric acid (BPUA-xx, indicated with yellow arrows), along with compressive Young’s moduli for other polymeric aerogels. Refer to [30] for material identification.

The model system for these studies was based on materials prepared using TIPM (see Figure 9 and Figure 10) and boric acid in DMF at room temperature (see Figure 4, reaction c), which yielded nanoporous polyurea networks that were dried with SCF CO_2_ to produce robust polyurea aerogels (referred to as BPUA-xx). BPUA-xx were chemically (by CHN, solid-state ^13^C NMR) and nanoscopically (by SEM, SAXS, N_2_ sorption) very similar to the reaction product of the same triisocyanate (TIPM) and water (referred to as PUA-yy; see Section 4.2 above). For example, the bulk densities of BPUA-xx and PUA-yy at the same total weight percentage of monomers in the sol (4–16% *w*/*w*) were 0.28–0.58 g·cm^−3^ and 0.39–0.60 g·cm^−3^, respectively. Microscopically, both materials consisted of assemblies of nanoparticles, as seen in PUA-yy aerogels of the same density range (see Figure 28). Minute differences were detected in the primary particle radii (6.2–7.5 nm for BPUA-xx vs. 7.0–9.0 nm for PUA-yy) and the micropore size within these primary particles (6.0–8.5 Å for BPUA-xx vs. 8.0–10 Å for PUA-yy). A significant difference was noted in the solid-state ^15^N NMR spectra, in which only PUA-yy showed some dangling -NH_2_ groups; no such -NH_2_ groups were detected in the spectra of BPUA-xx (Figure 31). Together, all data were consistent with exhaustive reaction of the isocyanate groups in BPUA-xx materials, in accordance with Figure 11. Exhaustive reactions removed dangling functional groups by stitching the ends of polymeric strands together, which is why BPUA-xx aerogels are the stiffest polymeric aerogels we are aware of at all densities (Figure 32). 

Residual boron in BPUA-xx aerogels was quantified with prompt gamma neutron activation analysis (PGNAA), and was found to be very low (about 0.05% *w*/*w*) and attributable to B_2_O_3_ according to ^11^B NMR [30]. Thus, any mechanism for systematic incorporation of boric acid into polymeric chains analogous to carboxylic acids was ruled out. In fact, it was derived mathematically that boron-terminated star polyurea from TIPM should contain ~3.3% *w*/*w* boron, *irrespective* of the size of the star monomer (refer to Appendix II in the Supporting Information of reference [30]). Upon reflection, it was concluded that it was fortuitous that the gelation study of isocyanates with mineral acids was conducted with H_3_BO_3_, whereas the resulting byproduct, B_2_O_3_, was easily removed from the resulting gels during post-gelation solvent exchanges, thereby leaving behind relatively easy-to-identify pure polyurea. With other mineral acids and with dense polymers that cannot be washed via solvent exchange, experimental results could have been misleading, as the corresponding oxides are insoluble and remain within the polymer (as confirmed via both skeletal density measurements and EDS). This being said, formation of polyurea with mineral acids other than boric acid provides a convenient method for in situ doping of mechanically robust porous polymeric networks and carbon aerogels derived from them, with a diverse array of oxides and pure metal nanoparticles (Au in the case of H_3_AuO_3_) with possible applications in catalysis. This method of doping polyurea and carbon aerogels with metallic and/or oxide nanoparticles remains open for further investigation.

### 4.4. Polyurea Aerogels as Random Copolymers with Polyamides and Polyimides

As mentioned in Section 2.4, isocyanates react with carboxylic acids to produce amides [32,33]. This reaction has been used extensively for the synthesis of polyamide aerogels [90,91,92,93,94]. However, when it was applied to the synthesis of polyamide aerogels with polyfunctional carboxylic acid that possess at least two carboxylic acid groups in relative positions that can yield intramolecular anhydrides (e.g., at two *ortho* positions of a phenyl ring, as in pyromellitic acid), the product turned out to be a mixture of the intended polyamide together with polyimide and polyurea, at roughly equal amounts.

**Figure 33:**
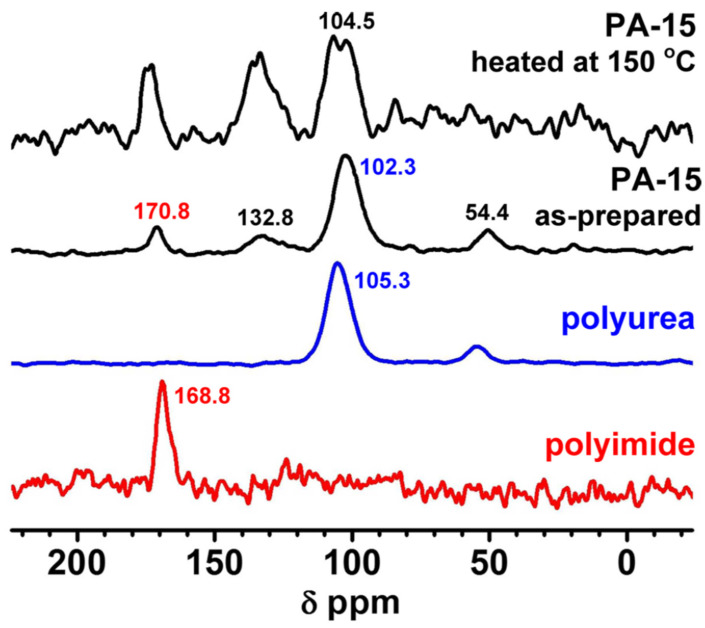
Solid-state CPMAS ^15^N NMR spectra of a representative as-prepared PA-xx aerogel before and after heating at 150 °C, together with the spectra of a TIPM/H_2_O-derived polyurea aerogel, and of a TIPM/pyromellitic-anhydride-derived polyimide aerogel, as indicated [95]. All spectra were referenced to liquid NH_3_.

**Figure 34:**
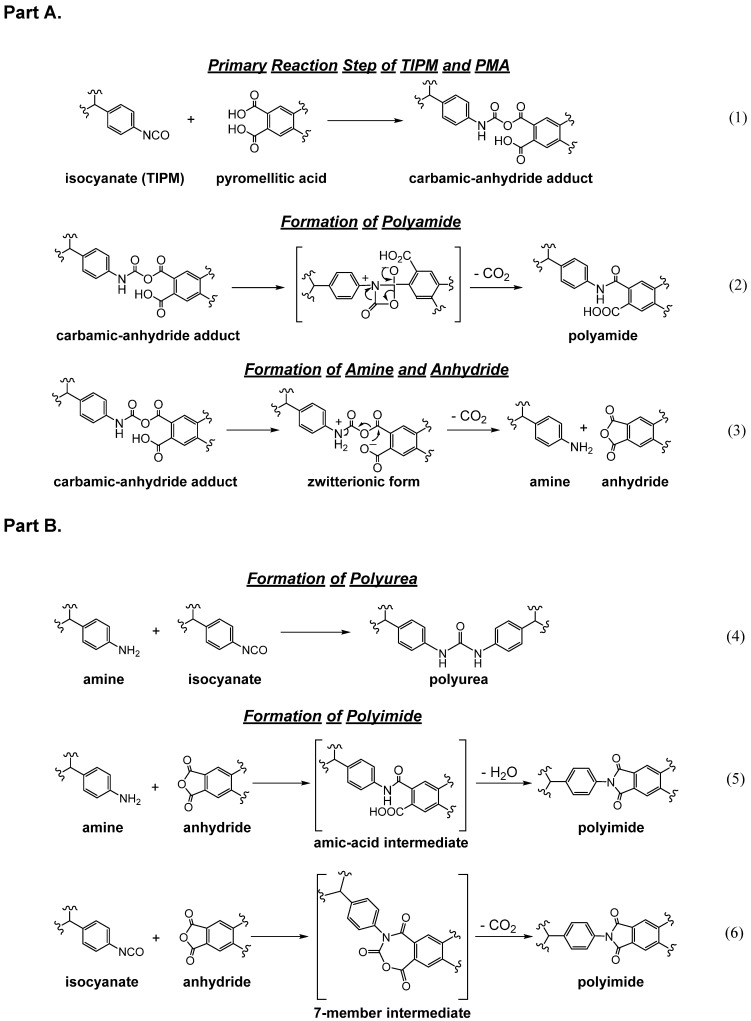
Proposed mechanism for the parallel formation of polyamide, polyurea, and polyimide from the reaction of an isocyanate and a 1,2-dicarboxylic acid [95,96]. **Part A**: Equations (1)–(3) describe the reactions and primary products following formation of the first reaction product of an isocyanate and a 1,2-dicarboxylic acid (a carbamic anhydride adduct). **Part B**: Equations (4)–(6) show the formation of the terminal products.

Specifically, polymeric aerogels (referred to as PA-xx) were prepared at room temperature via reaction of Desmodur RE (Figure 9) and pyromellitic acid in THF/ethyl acetate (EtOAc) mixtures [95,96]. The extension “xx” refers to the weight percentage of the monomers in the sol, and was set to 5, 10, 15, 20, and 25. Solid-state CPMAS ^15^N NMR (Figure 33) showed that the skeletal framework of as-prepared PA-xx consisted mainly of the carbamic anhydride adduct of the two reactants, which is the primary reaction product expected from the reaction of an isocyanate and a carboxylic acid (Figure 34, Equation (1)). According to well-understood processes occurring during the reaction of isocyanates and carboxylic acids [32,33], upon heating at 150 °C the carbamic anhydride adduct lost CO_2_ to produce either polyamide (Figure 34, Equation (2)) or an amine and an anhydride (Figure 34, Equation (3)). The last two intermediates had two options: the amine could react with still-unreacted isocyanate to produce polyurea (Figure 34, Equation (4)—according to Figure 5), or it could react with the anhydride with which it was produced simultaneously (see Figure 34, Equation (3)) to give polyimide, according to the conventional method of polyimide synthesis (Figure 34, Equation (5)) [10]. Polyimide was also produced via reaction of the anhydride with yet-unreacted isocyanate (Figure 34, Equation (6)) [97,98,99,100]. Overall, according to Figure 34, the aerogel consisted of a copolymer of polyamide, polyurea, and polyimide, in agreement with the solid-state ^15^N NMR spectrum at the top of Figure 33 [95,96]. 

Stepwise pyrolytic decomposition of the random polyurea–polyamide–polyimide copolymer yielded carbon aerogels with both open and closed microporosity [95,96]. The open-micropore surface area increased from < 15 m^2^·g^−1^ in PA-xx to 340 m^2^·g^−1^ in the corresponding carbon aerogels. Reactive etching (also referred to as activation) of the carbon aerogels with CO_2_ at 1000 °C opened access to the closed pores, and the BET surface area reached 1750 m^2^·g^−1^, out of which 1150 m^2^·g^−1^ was allocated to micropores. At 0 °C, such pyrolytically activated carbon aerogels demonstrated a good balance of adsorption capacity for CO_2_ (up to 4.9 mmol·g^−1^) and selectivity toward other gases (calculated by applying Henry’s law on the appropriate isotherms). The selectivity toward CO_2_ versus H_2_ (up to 928:1) is suitable for pre-combustion fuel purification. With respect to post-combustion CO_2_ capture and sequestration (CCS), the selectivity toward CO_2_ versus N_2_ was in the 17:1 to 31:1 range. To explain the high uptake of CO_2_, in addition to the typical factors involved in gas sorption (kinetic diameters, quadrupole moments, and polarizability of the adsorbates), it was also suggested that CO_2_ is first engaged by surface pyridinic and pyridonic N on carbon (identified with XPS), and then CO_2_ keeps on accumulating on already-adsorbed CO_2_ as a result of an energy-neutral surface reaction (Equation (1)) until the micropores are filled. Relatively high uptake of CH_4_ (2.16 mmol g^−1^ at 0 °C/1 bar) was attributed to its low polarizability. Overall, high CO_2_ selectivity, in combination with attractive CO_2_ adsorption capacities, low monomer cost, and the innate physicochemical stability of carbon, rendered the materials of this study a point of departure for subsequent studies by the same authors, where the CO_2_ uptake was elevated to ~15 mmol·g^−1^ [101].

pyridinic/pyridonic-N^+^−(C=O)O^−^ + CO_2_ ---> pyridinic/pyridonic-N^+^−(C=O)O−(C=O)O^−^ (1)

### 4.5. Polyurea-Crosslinked Oxide and Biopolymer Aerogels

**Figure 35:**
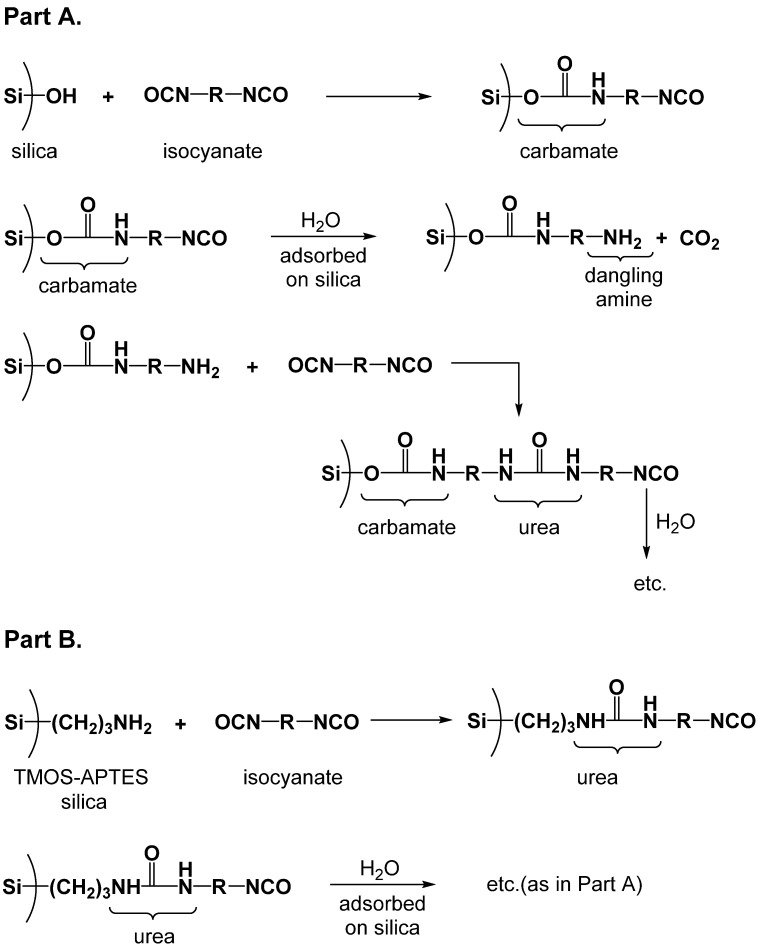
Crosslinking with isocyanates of: (**Part A**: Native alkoxide-derived silica; **Part B**: Amine-modified silica (e.g., obtained via co-gelation of TMOS and APTES; see text). For simplicity, the isocyanate is shown as a diisocyanate.

**Figure 36:**
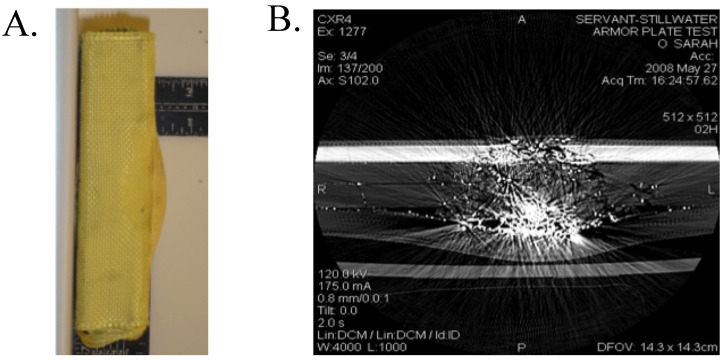
(**A**) A 15.2 cm × 15.2 cm × 3.6 cm (6” × 6” × 1.4”) armor plate incorporating three 0.5” thick panels of polyurea-crosslinked surfactant-templated silica aerogel as the energy-absorbing layer, SiC as a front plate, and Kevlar cloth for wrapping. The armor plate stopped a 0.308 Winchester round (NIJ Level-III threat) fired with a Remington 700 police sniper rifle from 15.2 m (50 feet, bullet entry point from the left). (**B**) Computed microtomography showing the bullet stopped after the second aerogel panel (bullet entry point from the top). The backside deformation of the target plate was 1.27 cm (0.5”, middle-right surface) [103].

As stated in the Introduction, understanding the mechanism of crosslinking silica aerogels with multifunctional isocyanates led to the developments described in Section 4.2 of this article. In other words, polymeric polyurea aerogels and polyurea-crosslinked aerogels are intimately related. It should be noted that “polymer crosslinking” in the context of aerogels refers to a nanoscale phenomenon, where nanosized skeletal building blocks are bridged with polymer. For this, suitable monomers bond chemically to functional groups on the surface of the skeletal framework, and subsequently build tethers between those binding sites. *Part A* in Figure 35 summarizes the crosslinking process of silica with isocyanates [102], but that process also applies to other –OH-bearing surfaces, such as those of biopolymer aerogels (see below). In brief, a multifunctional isocyanate (typically trifunctional) first forms a urethane linkage to the surface of a preformed silica wet gel via reaction with dangling –OH groups, but the intersite bridges of skeletal particles consist of polyurea formed first by hydrolysis of dangling isocyanates from gelation water remaining adsorbed on the surface of the wet gel, and subsequent reaction of the newly formed amines with more isocyanate in the pores (see Figure 8). The net result is an oxide skeletal framework coated conformally with a nanothin layer of polyurea. The most remarkable feature of these materials is their mechanical strength, which renders them suitable even for ballistic protection (i.e., armor; see Figure 36) [103]. 

**Figure 37:**
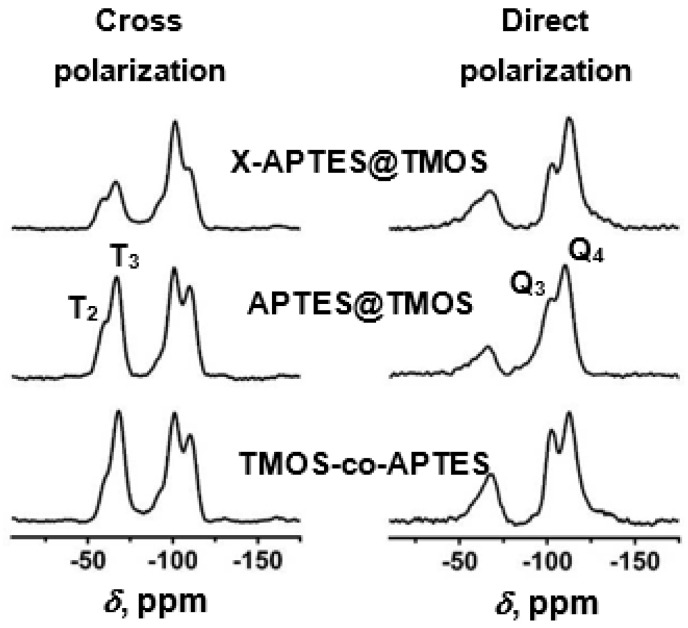
Solid-state MAS ^29^Si NMR spectra of aerogels obtained under direct and cross-polarization (CP). The difference in intensity of certain resonances indicates proximity to protons. TMOS-co-APTES: via co-gelation of TMOS and APTES; APTES@TMOS: APTES was added post-gelation to the TMOS wet gel (for uniform reaction with the particles, the TMOS wet gel was in the form of powder); X-APTES@TMOS: APTES@TMOS silica sample crosslinked with polyurea derived from Desmodur N3200 (Figure 7). Note that (a) TMOS-co-APTES and APTES@TMOS gave identical spectra, confirming that APTES reacts with the surface of the TMOS network, which is formed fist; (b) Q_3_ and T_3_ under CP are more intense because of their proximity to protons; and (c) after crosslinking, T_3_ remained unaffected because it was already in the vicinity of the –CH_2_– protons of APTES, but the intensity of Q_3_ increased significantly, supporting the view that crosslinking took place not only via the –NH_2_ groups coming from APTES, but also via the –OH groups of Q_3_-silica [104].

The next step in the development of polyurea-crosslinked aerogels was to enforce urea formation, starting from the surface of the solid network. This was achieved either via co-gelation of tetramethyl orthosilicate (TMOS) and 3-aminopropyl triethoxysilane (APTES), or via post-gelation modification of a TMOS-derived gel with APTES. Both processes yielded the same material—a silica network, surface-modified with amines. A multifunctional isocyanate introduced in the pores of the newly prepared wet gels reacted quickly with dangling surface amines from APTES, followed by reaction with gelation water remaining adsorbed at the surface of the silica, leading to a silica network crosslinked with polyurea starting from the surface of the silica (*Part B* in Figure 35). Those bonding relationships were elucidated with solid-state ^29^Si NMR (Figure 37) [104].

**Figure 38:**
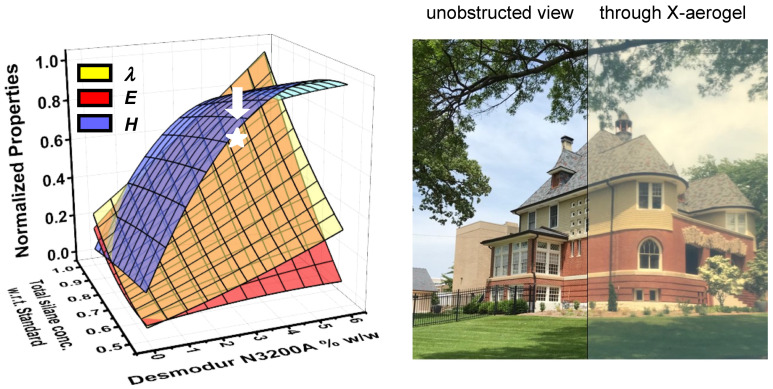
**Left**: 3D plot of Young’s modulus (*E*), thermal conductivity (*λ*), and total haze (*H*) of polymer-crosslinked silica aerogels normalized to their respective highest values. The arrow marks the composition of the sample used in the photograph on the right. **Right**: view through a 7 mm thick optimized polymer-crosslinked (X-) silica aerogel panel with the combination of properties shown by the arrow on the left (*ρ*_b_ = 0.329 ± 0.003 g·cm^−3^; porosity = 76% *v*/*v*; BET surface area: 330 m^2^·g^−1^; *E* = 70 ± 3 MPa; *λ* = 22.5 ± 0.8 mW·m^−1^·K^−1^; bulk haze at 3 mm = 9.6) [106].

TMOS–APTES-derived silica aerogels crosslinked with Desmodur N3300A (but also with Desmodur N3200) are translucent and colorless [105]. A recent design-of-experiments (DoE) study sought to optimize strength with minimal haze and minimal thermal conductivity for window applications; Figure 38 shows the results [106]. 

**Figure 39:**
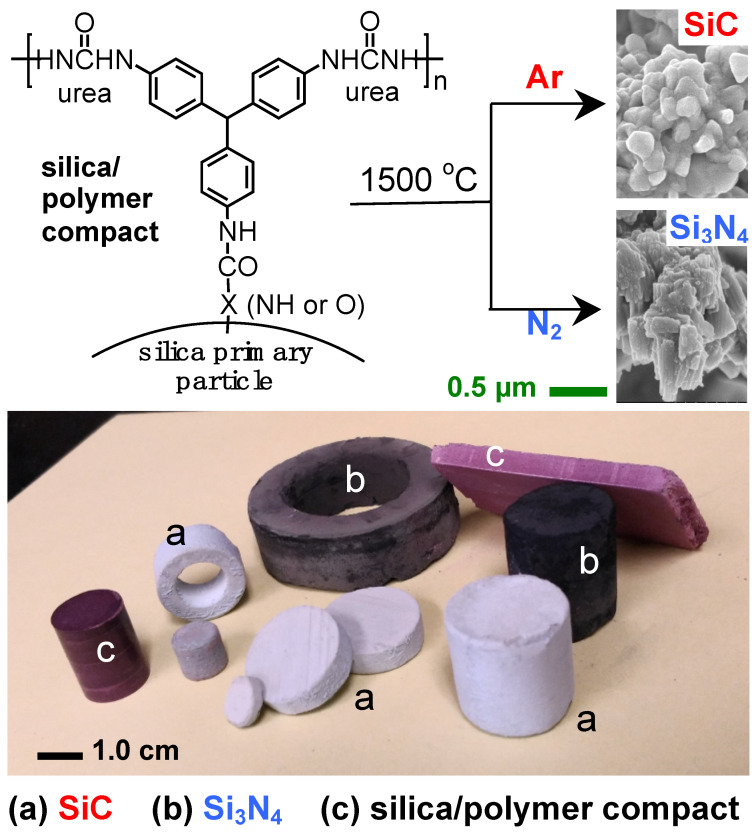
**Top**: nanoparticles of an APTES@TMOS aerogel network crosslinked with carbonizable polyurea derived from Desmodur RE (Figure 9). Pyrolysis at 1500 °C under Ar yields pure SiC aerogels, while at 1500 °C under N_2_ it yields pure Si_3_N4 aerogels. **Bottom**: photographs of SiC and Si_3_N_4_ aerogel monoliths with different form factors prepared from silica/polymer compacts (also included in the photograph) according to the method on top [104].

Another application of polyurea-crosslinked oxide aerogels is as starting materials for purely metallic and ceramic aerogels. For those applications, the crosslinking polyurea has to be carbonizable, and a good monomer for this is Desmodur RE (Figure 9). SiC and Si_3_N_4_ aerogels were prepared carbothermally at 1500 °C under Ar or N_2_, respectively, via reaction of the silica backbone of polyurea-crosslinked silica xerogel powders compressed into desirable shapes before pyrolysis. Figure 39 shows monolithic SiC and Si_3_N_4_ aerogels with different form factors prepared via this method [104]. 

**Figure 40:**
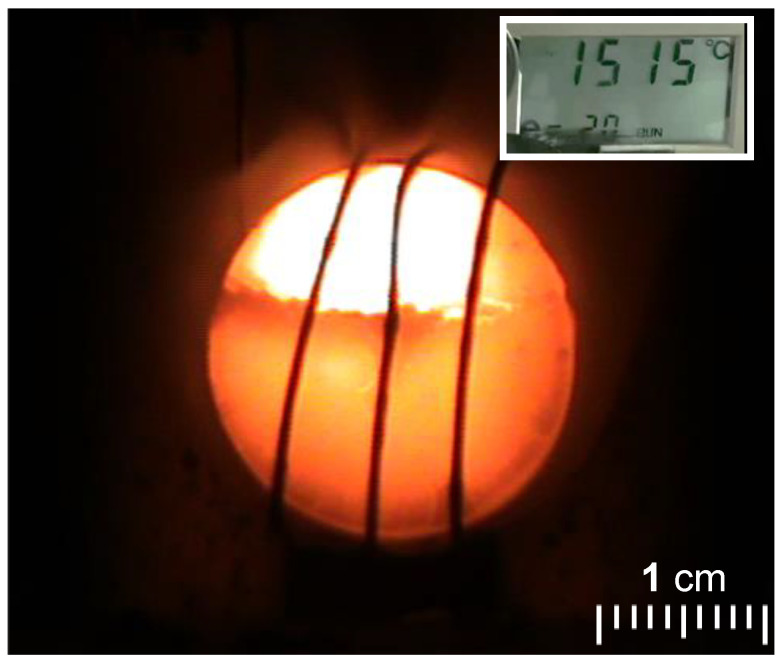
LiClO_4_-infiltrated Co(0) aerogel disc (21.8 mm in diam., 3.7 mm thick) ignited with an electric resistor wrapped around the disc. *Inset:* display of an optical pyrometer showing the temperature at the center of the disc reaching 1515 °C within 4 s after ignition [107].

If the stoichiometric balance of silica to Desmodur RE favors the formation of carbon, then this monolithic aerogel-via-xerogel powder method can be used for the preparation of carbon aerogels without the need for a supercritical drying step. In a parallel effort, Desmodur-RE-crosslinked cobaltia xerogel powders were compressed into discs that were converted carbothermally into pure metallic cobalt aerogels. The latter were melt-infiltrated with LiClO_4_ and were operated as thermites; when ignited with a resistor, combustion of Co(0) reached temperatures in excess of 1500 °C (Figure 40) [107].

**Figure 41:**
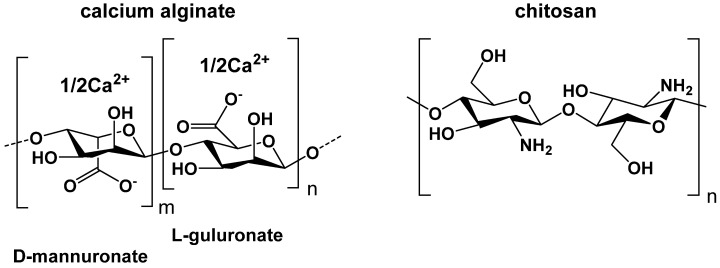
The chemical composition of the skeletal frameworks of calcium alginate and chitosan aerogels.

Lately, the polyurea-crosslinking process has been found to be a very fertile ground in the synthesis of mechanically strong biopolymer aerogels consisting of two types of polysaccharides: calcium alginate, or chitosan (Figure 41). Both types of biopolymer aerogels come from wet gels prepared from aqueous sols; therefore, their surfaces are rich in adsorbed gelation water. In this way, hydroxyl-group-rich preformed alginate frameworks have been crosslinked with polyurea, as shown in *Part A* in Figure 35, while preformed chitosan wet gels with surfaces rich in both –OH and –NH_2_ groups have been crosslinked with polyurea, just like APTES-modified silica, as shown in *Part A* and *Part B* in Figure 35. Specifically, monolithic calcium alginate aerogels consisting of two different guluronate-to-mannuronate ratios were crosslinked with aliphatic triisocyanate Desmodur N3300A into hydrophobic, extremely mechanically strong X-alginate aerogel monoliths in analogy to silica [108]. 

**Figure 42:**
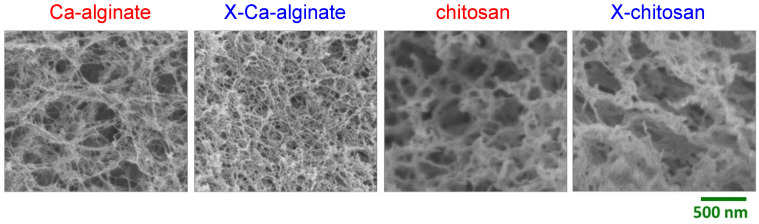
Representative SEM images from the interior of native biopolymer and X-biopolymer aerogel beads, as indicated. Scale bar in all cases = 500 nm (magnification: 50,000×) [109].

Similarly, both X-alginate and X-chitosan aerogel beads were also prepared by crosslinking the corresponding native wet-gel beads with Desmodur N3300A [109]. The resulting crosslinked beads were robust, and retained the fibrillar morphology of their native counterparts (Figure 42) [109]. The *K*-index of X-alginate aerogels was in the range of 1.0–1.6, and followed the morphological trends established with pure Desmodur-N3300A-derived polyurea aerogels (see Figure 26) [108]. A more detailed study of the skeletal framework of X-alginate aerogels crosslinked with either Desmodur N3300A or Desmodur RE using small-angle neutron scattering (SANS) showed that the fibers of the alginate network consisted of primary particles 8.3 nm in radius that grew to 10.0 nm in radius upon crosslinking [110]. Aerogel fibers consisting of particles have been observed in polyurea and polyimide aerogels before, and in both cases neutron scattering has proven to be the technique of choice for the elucidation of their morphology [20,97]. 

**Figure 43:**
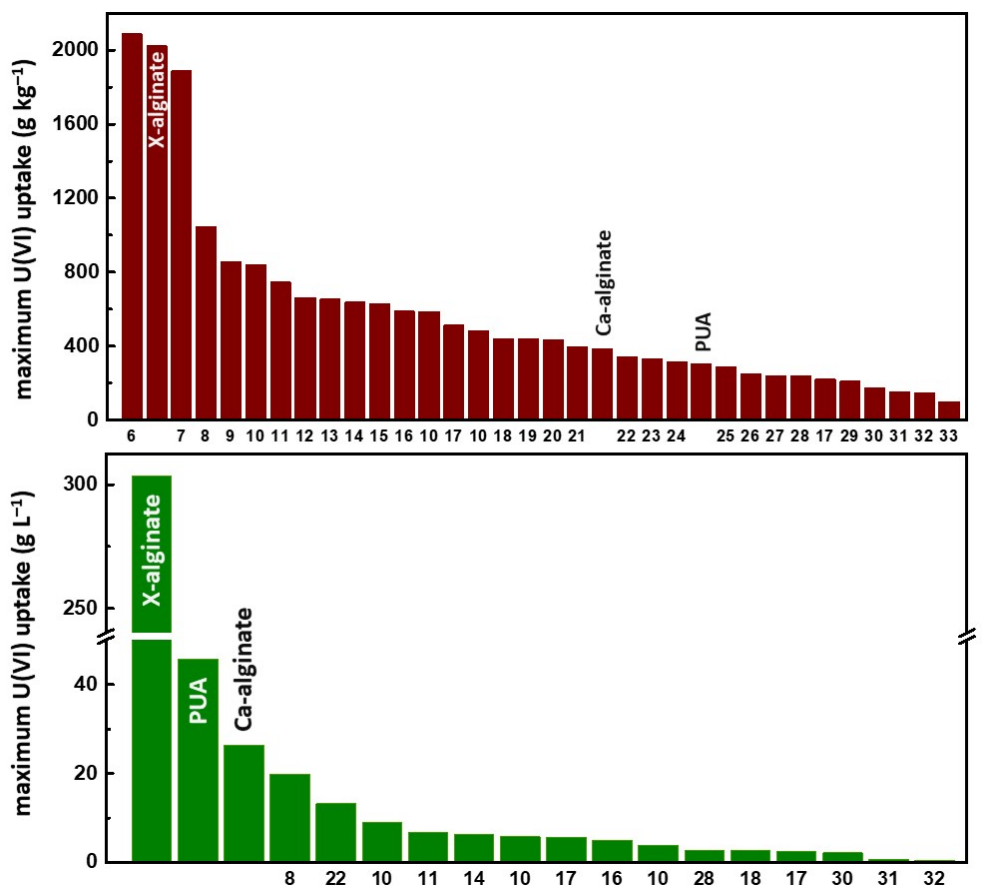
Maximum U(VI) sorption capacity from laboratory solutions using various aerogel materials. **Top**: maximum U(VI) uptake per weight of adsorbent. **Bottom**: maximum U(VI) uptake per volume of adsorbent. Numbers along the horizontal axes refer to the corresponding references in the original publication [112]. For comparison, results from control experiments using native Ca alginate aerogels and Desmodur-RE-derived polyurea aerogels (PUAs) are also included for reference.

A promising application of calcium alginate aerogel beads (3.3 mm in diameter) crosslinked with rigid aromatic Desmodur RE triisocyanate has been demonstrated in seawater decontamination from metal ions, organic solvents, and oils [111]. Specifically, crosslinked calcium alginate aerogel beads, 90% porous, consisting of 54% *w*/*w* polyurea and 2% calcium with a BET surface area of 459 m^2^·g^−1^, adsorb Pb(II) not only from ultrapure water (29 mg·g^−1^), but also from seawater (13 mg·g^−1^), with high selectivity toward other ions. It was proposed that the Pb(II)-uptake mechanism involves replacement of Ca(II) by Pb(II) ions coordinated with the carboxylate groups of the alginate backbone. After treatment with a sodium–EDTA solution, the beads were reused at least twice, without significant loss of activity. These X-Ca alginate aerogel beads could also uptake organic solvents and oils from seawater; the volume of the adsorbate could be as high as the total pore volume of the aerogel (6.0 mL·g^−1^), and the absorption was complete within seconds [111]. In an interesting extension of this work, the same type of Desmodur-RE-crosslinked calcium alginate aerogel beads could remove uranium(VI) from laboratory and environmental waters, with an extremely high adsorption capacity for uranium(VI) (up to 2023 g·kg^–1^ of aerogel) [112]. The adsorption process was endothermic, entropy-driven, and followed the *Langmuir* isotherm model. FTIR spectroscopic data indicated that adsorption occurs via formation of inner-sphere complexes between the surface functional groups of X-alginate beads and UO_2_^2+^. The post-adsorption presence of uranium in the adsorbent was confirmed and quantified via EDS analysis. Compared to other aerogel adsorbents of UO_2_^2+^ from the literature, X-alginate aerogels showed one of the highest sorption capacities per weight, and the highest per volume (Figure 43). Uranium could be recovered almost quantitatively (~100%) in aqueous solutions of Na_2_CO_3_ (pH 11) or EDTA (pH 10). X-calcium alginate aerogels were also effectively applied for the removal of uranium(VI) from acid mine drainage (AMD), groundwater, and seawater samples. The X-alginate aerogel beads were stable in all of the above environments (i.e., no swelling, shrinking, or disintegration was observed). It was concluded that the extraordinary adsorption capacity—even in the presence of competing metal cations—and stability of the X-alginate aerogel beads in environmental waters render them excellent candidates for uranium(VI) removal from aqueous environments [112].

Finally, various metal-doped polyurea-crosslinked alginate aerogel beads (X–M–alginate; M: Co, Ni, Cu) were prepared in the same fashion as the calcium alginate beads, and were crosslinked with Desmodur RE [113]. The X–M–alginate aerogels consisted of 49–63% polyurea and contained 2–7% metal ions, and they were fibrous macro/meso/microporous materials with porosities up to 94% *v*/*v*, and BET surface areas in the 245–486 m^2^·g^−1^ range, comparable to those of native M–alginate aerogels (258–542 m^2^·g^−1^). The pyrolysis of X–M–alginate aerogels (M: Co, Ni, Cu) at 800 °C yielded carbon aerogels (X–M–C; 33–37% yield) doped with the corresponding metals (as well as with Cu_2_O in the case of X–Cu–C), with crystallite sizes of around 22 nm. The X–M–C aerogels retained the general fibrous morphology of their precursor aerogels (X–M–alginate), while all identifiable differences were considered to be indicative of the effect of the metal on the nanostructure of the corresponding carbon. The porosities of all X–M–C aerogels were in the range of 88–92% *v*/*v*, including macro-, meso-, and micropores. Their BET surface areas were in the range of 426–541 m^2^·g^−1^, of which 208–319 m^2^·g^−1^ was allocated to micropores. In addition to the metals, XPS, Raman, and FTIR analyses showed the presence of oxygen and nitrogen functionalities. Carbon in the X–M–C aerogels showed signs of stacking of graphene oxide sheets (14–15 nm), but also a low degree of graphitization with a large number of defects. That work was considered by the authors as a direct and inexpensive method for the preparation of fibrous metal-, oxygen-, and nitrogen-doped carbon aerogels with the potential for catalytic and electrochemical applications [113].

## 5. Conclusions

Over the last 10 years, polyurea aerogels have emerged as an important class of aerogels, with both fundamental and practical significance. In terms of fundamentals, polyurea aerogels have become the point of departure for the development of new molecular-level chemical understanding of the reaction of isocyanates with mineral acids and with polyfunctional carboxylic acids. A major part in those studies was played by material characterization via solid-state ^15^N NMR, the use of which will only be expanded in the future. At the nanoscopic level, polyurea aerogels continue to play an important role in terms of dimensional stabilization of mechanically weak skeletal frameworks, such as those of inorganic oxides and, more recently, of biopolymer aerogels from alginate and chitosan. The latter line of research is currently in its infancy, and is expected to prove itself extremely useful when applied to other classes of biopolymers beyond sugars—for example, to proteins, nucleic acids, etc. Still, at the nanoscopic level, polyurea aerogels display a very broad range of nanomorphologies—most remarkably within the same chemical composition; thus, they have become a powerful platform for the study of nanostructured matter in the spirit of Kistler’s original invention and early work. More specifically, the extremely broad range of nanomorphologies of polyurea aerogels has raised the question of whether complex nanostructures can be expressed quantitatively, leading in turn to the question of whether such geometric complexity could be designed a priori. The result of this line of reasoning was the development of the *K*-index, which successfully addressed both of these questions. In terms of synthesis, the development and implementation of more environmentally benign (“greener”) chemistries will be a boon to the commercial uses of polyurea aerogels. “Green” chemistry can be approached by substitution of isocyanates with other reagents, by substitution of organic solvents with water, or by substitution of both. Efforts to eliminate isocyanates might involve CO_2_ [114] or carbonates [115]. Efforts to implement gelation in water might, for example, involve synthesis through *N*-substituted urea intermediates obtained from potassium isocyanate and primary or secondary amines [116]. 

As reviewed in this article, polyurea aerogels have been demonstrated for applications in thermal and acoustic superinsulation, ballistic protection, blast-wave mitigation, and oil-spill cleanup. Polyurea-crosslinked oxide aerogels are the point of departure for ceramic and metallic aerogels, while recently developed polyurea-crosslinked alginate aerogels are prime candidates for application in the decontamination of natural waters from heavy metals (e.g., Pb, U). In addition, functional carbon aerogels produced from polyurea and polyurea–copolymer aerogels are expected to find uses as electrodes for electrochemical energy-storage and production systems (e.g., batteries, capacitors, fuel cells), as media for pre- and post-combustion sequestration of CO_2_ in power plants, and as catalyst supports. 

Owing to their versatility and desirable combination of material properties, polyurea aerogels have become commercially available. The future of these materials in academia and industry is undoubtedly bright, as advanced polyurea and polyurea-crosslinked aerogels are well poised to address and resolve many of society’s current and emerging challenges related to energy, healthcare, and the environment.

## Figures and Tables

**Table 1:** Relative reactivity of active hydrogen nucleophiles based on N and O toward –N=C=O [21]. Formulae in parentheses emphasize the nucleophilic centers.

Active-Hydrogen Nucleophile	Relative Reaction Rates (Uncatalyzed at 25 °C)	Product Classification
Primary aliphatic amines (R–NH_2_)	100,000	Urea
Secondary aliphatic amines (R–NH–R’)	20,000–50,000	Urea
Primary aromatic amines (Ar–NH_2_)	200–300	Urea
Primary hydroxyl (e.g., R–OH)	100	Urethane
Water (H–OH)	100	Urea
Carboxylic acids (R(C=O)–OH)	40	Amide
Secondary hydroxyls (e.g., RCH(OH)R’)	30	Urethane
Ureas (–NH(C=O)NH–)	15	Biuret
Tertiary hydroxyls (R_3_C–OH)	0.5	Urethane
Urethane (–NH(C=O)O–)	0.3	Allophanate

## References

[1] Leventis N., Sadekar A., Chandrasekaran N., Sotiriou-Leventis C. (2010). Click synthesis of monolithic silicon carbide aerogels from polyacrylonitrile-crosslinked 3D silica networks. Chem. Mater..

[2] Vareda J.P., Lamy-Mendes A., Durães L. (2018). A reconsideration on the definition of the term aerogel based on current drying trends. Microporous Mesoporous Mater..

[3] Kistler S.S. (1931). Coherent expanded aerogels and jellies. Nature.

[4] Pierre A.C., Pajonk G.M. (2002). Chemistry of aerogels and their applications. Chem. Rev..

[5] Long J.W., Swider-Lyons K.E., Stroud R.M., Rolison D.R. (2000). Design of pore and matter architectures in manganese oxide charge-storage materials. Electrochem. Solid-State Lett..

[6] Mansour A.N., Smith P.H., Baker W.M., Balasubramanian M., McBreen J. (2003). A comparative in situ X-ray absorption spectroscopy study of nanophase V_2_O_5_ aerogel and ambigel cathodes. J. Electrochem. Soc..

[7] Mandal C., Donthula S., Rewatkar P.M., Sotiriou-Leventis C., Leventis N. (2019). Experimental deconvolution of depressurization from capillary shrinkage during drying of silica wet-gels with SCF CO_2_-Why aerogels shrink?. J. Sol-Gel Sci. Technol..

[8] Kistler S.S. (1932). Coherent expanded aerogels. J. Phys. Chem..

[9] Biesmans G., Randall D., Francais E., Perrut M. (1998). Polyurethane-based organic aerogels’ thermal performance. J. Non-Cryst. Solids.

[10] Rhine W., Wang J., Begag R. (2006). Polyimide Aerogels, Carbon Aerogels, and Metal Carbide Aerogels and Methods of Making Same. U.S. Patent.

[11] Lee J.K., Gould G.L. (2007). Polydicyclopentadiene based aerogel: A new insulation material. J. Sol-Gel Sci. Technol..

[12] De Vos R., Biesmans G.L.J.G. (1996). Organic Aerogels. U.S. Patent.

[13] Lorjai P., Chaisuwan T., Wongkasemjit S. (2009). Porous structure of polybenzoxazine-based organic aerogel prepared by sol-gel process and their carbon aerogels. J. Sol-Gel Sci. Technol..

[14] An H., Wang Y., Wang X., Zheng L., Wang X., Yi L., Bai L., Zhang X. (2010). Polypyrrole/carbon aerogel composite materials for supercapacitor. J. Power Sources.

[15] Leventis N. (2007). Three Dimensional Core-Shell Superstructures: Mechanically Strong Aerogels. Acc. Chem. Res..

[16] Leventis N., Vassilaras P., Fabrizio E.F., Dass A. (2007). Polymer nanoencapsulated rare earth aerogels: Chemically complex but stoichiometrically similar core-shell superstructures with skeletal properties of pure compounds. J. Mater. Chem..

[17] Leventis N., Sotiriou-Leventis C., Mulik S., Dass A., Schnobrich J., Hobbs A., Fabrizio E.F., Luo H., Churu G., Zhang Y. (2008). Polymer nanoencapsulated mesoporous vanadia with unusual ductility at cryogenic temperatures. J. Mater. Chem..

[18] Leventis N., Sotiriou-Leventis C., Zhang G., Rawashdeh A.-M.M. (2002). Nano Engineering Strong Silica Aerogels. NanoLetters.

[19] Leventis N., Mulik N.S., Sotiriou-Leventis C. (2008). Macroporous electrically conducting carbon networks by pyrolysis of isocyanate cross-linked resorcinol-formaldehyde aerogels. Chem. Mater..

[20] Leventis N., Sotiriou-Leventis C., Chandrasekaran N., Mulik S., Larimore Z.L., Lu H., Churu G., Mang J.T. (2010). Multifunctional polyurea aerogels from isocyanates and water. A structure-property case study. Chem. Mater..

[21] Lee S.T., Ramesh N.S. (2004). Polymeric Foams: Mechanism and Materials.

[22] Saunders J.H., Frisch K.C. (1964). Polyurethane Chemistry and Technology: Chemistry.

[23] Stevens M.P. (1990). Polymer Chemistry. An Introduction.

[24] Davis T.L., Ebersole F. (1934). Relative velocities of reaction of amines with phenyl isocyanate. J. Am. Chem. Soc..

[25] Neumann W., Fischer P. (1962). Carbodiimide aus Isocyanaten. Angew. Chem..

[26] https://coatings.specialchem.com/product/r-covestro-desmodur-n-3200.

[27] Wicks Z.W., Jones F.N., Pappas S.P., Wicks D.A. (2007). Organic Coatings, Science & Technology.

[28] Odian G. (2004). Principles of Polymerization.

[29] Dodge J., Rogers M.E., Long T.E. (2003). Polyurethanes and Polyureas. Synthetic Methods in Step-Growth Polymers.

[30] Leventis N., Sotiriou-Leventis C., Saeed A.M., Donthula S., Majedi Far H., Rewatkar P.M., Kaiser H., Robertson J.D., Lu H., Churu G. (2016). Nanoporous polyurea from a triisocyanate and boric acid: A paradigm of a general reaction pathway for isocyanates and mineral acids. Chem. Mater..

[31] Aries R.S. (1960). Polymers from Boric Acid and Organic Diisocyanates. U.S. Patent.

[32] Xiao H., Xiao H.X., Frisch K.C., Malwitz N. (1994). Kinetic studies of the reactions between isocyanates and carboxylic acids. High Perform. Polym..

[33] Sorenson W.R. (1959). Reaction of an isocyanate and a carboxylic acid in dimethyl sulfoxide. J. Org. Chem..

[34] Trappe V., Prasad V., Cipelletti L., Segre P.N., Weitz D.A. (2001). Jamming phase diagrams for attractive particles. Nature.

[35] Lu P.J., Zaccarelli E., Ciulla F., Schofield A.B., Sciortino F., Weitz D.A. (2008). Gelation of particles with short-range attraction. Nature.

[36] Rouwhorst J., Ness C., Stoyanov S., Zaccone A., Schall P. (2020). Nonequilibrium continuous phase transition in colloidal gelation with short-range attraction. Nature Commun..

[37] Bang A., Buback C., Sotiriou-Leventis C., Leventis N. (2014). Flexible aerogels from hyperbranched polyurethanes: Probing the role of molecular rigidity with poly(urethane acrylates) versus poly(urethane norbornenes). Chem. Mater..

[38] Papastergiou M., Kanellou A., Chriti D., Raptopoulos G., Paraskevopoulou P. (2018). Poly(urethane-acrylate) aerogels via radical polymerization of dendritic urethane-acrylate monomers. Materials.

[39] Mohite D.P., Larimore Z.J., Lu H., Mang J.T., Sotiriou-Leventis C., Leventis N. (2012). Monolithic hierarchical fractal assemblies of silica nanoparticles cross-linked with polynorbornene via ROMP: A structure-property correlation from molecular to bulk through nano. Chem. Mater..

[40] Mohite D.P., Mahadik-Khanolkar S., Luo H., Lu H., Sotiriou-Leventis C., Leventis N. (2013). Polydicyclopentadiene aerogels grafted with PMMA: II. Nanoscopic characterization and origin of macroscopic deformation. Soft Matter.

[41] Chidambareswarapattar C., McCarver P.M., Luo H., Lu H., Sotiriou-Leventis C., Leventis N. (2013). Fractal multiscale nanoporous polyurethanes: Flexible to extremely rigid aerogels from multifunctional small molecules. Chem. Mater..

[42] Malakooti S., ud Doulah A.B.M.S., Ren Y., Kulkarni V.N., Soni R.U., Edlabadkar V.A., Zhang R., Vivod S.L., Chariklia Sotiriou-Leventis C., Leventis N. (2021). Meta-aerogels: Auxetic shape-memory polyurethane aerogels. ACS Appl. Polym. Mater..

[43] Członka S., Bertino M.F., Kośny J., Shukla N. (2018). Freeze-drying method as a new approach to the synthesis of polyurea aerogels from isocyanate and water. J. Sol-Gel Sci. Technol..

[44] Steiner S.A., Griffin J.S., Wunsch B.H., Schneider J.N. (2020). Systems and Methods for Producing Aerogel Materials. U.S. Patent.

[45] Lee J.K., Gould G.L., Rhine W. (2009). Polyurea based aerogels for a high performance thermal insulation material. J. Sol-Gel Sci. Technol..

[46] https://azelisamericascase.com/wp-content/uploads/2020/03/JEFFAMINE-amines-Brochure.pdf.

[47] https://solutions.covestro.com/en/products/multranol/multranol-9185_05213088-12942099?SelectedCountry=US.

[48] Trifu R., Gould G., White S. (2017). Flexible polyisocyanate based aerogels. MRS Adv..

[49] Shinko A., Jana S.C., Meador M.A. (2015). Crosslinked polyurea aerogels with controlled porosity. RSC Adv..

[50] Shinko A., Jana S.C., Meador M.A. (2018). Crosslinked polyurea-co-polyurethane aerogels with hierarchical structures and low stiffness. J. Non-Cryst. Solids.

[51] Wu X., Wu Y., Zou W., Wang X., Du A., Zhang Z., Shen J. (2019). Synthesis of highly cross-linked uniform polyurea aerogels. J. Supercrit. Fluids.

[52] Lu X., Caps R., Fricke J., Alviso C.T., Pekala R.W. (1995). Correlation between structure and thermal conductivity of organic aerogels. J. Non-Cryst. Solids.

[53] Lu X., Nilsson O., Fricke J., Pekala R.W. (1993). Thermal and electrical conductivity of monolithic carbon aerogels. J. Appl. Phys..

[54] Chriti D., Raptopoulos G., Papastergiou M., Paraskevopoulou P. (2018). Millimeter-size spherical polyurea aerogel beads with narrow distribution. Gels.

[55] Petricevic R., Glora M., Fricke J. (2001). Planar fibril reinforced carbon aerogels for application in PEM fuel cells. Carbon.

[56] Petricevic R., Glora M., Möginger A., Fricke J. (2001). Skin formation on RF aerogel sheets. J. Non-Cryst. Solids.

[57] http://www.aerogeltechnologies.com/airloy/products.

[58] Leventis N., Sotiriou-Leventis C., Mulik S. (2019). Three-Dimensional Porous Polyurea Networks and Methods of Manufacture. U.S. Patent.

[59] American Society for Metals (1998). ASM Engineering Materials Handbook, Composites Volume 1.

[60] Jones S.M. (2007). A method for producing gradient density aerogel. J. Sol-Gel Sci. Technol..

[61] Leventis N., Sotiriou-Leventis C., Chandrasekaran N., Mulik S., Chidambareswarapattar C., Sadekar A., Mohite D., Mahadik S.S., Larimore Z.J., Lu H. (2011). Isocyanate-derived organic aerogels: Polyureas, polyimides, polyamides. Mater. Res. Soc. Symp. Proc. Mater. Res. Soc..

[62] Bian Q., Chen S., Kim B.-T., Leventis N., Lu H., Chang Z., Lei S. (2011). Micromachining of polyurea aerogel using femtosecond laser pulses. J. Non Cryst. Solids.

[63] Leventis N., Chidambareswarapattar C., Bang A., Sotiriou-Leventis C. (2014). Cocoon-in-web-like superhydrophobic aerogels from hydrophilic polyurea and use in environmental remediation. ACS Appl. Mater. Interfaces.

[64] Weigold L., Mohite D.P., Mahadik-Khanolkar S., Leventis N., Reichenauer G. (2013). Correlation of microstructure and thermal conductivity in nanoporous solids: The case of polyurea aerogels synthesized from an aliphatic tri-isocyanate and water. J. Non-Cryst. Solids.

[65] Mohite D.P., Mahadik-Khanolkar S., Luo H., Lu H., Sotiriou-Leventis C., Leventis N. (2013). Polydicyclopentadiene aerogels grafted with PMMA: I. Molecular and interparticle crosslinking. Soft Matter.

[66] Fricke J., Lu X., Wang P., Buettner D., Heinemann U. (1992). Optimization of monolithic silica aerogel insulants. Int. J. Heat Mass Transf..

[67] Gibson L.J., Ashby M.F. (1999). Cellular Solids—Structure and Properties.

[68] Weigold L., Reichenauer G. (2014). Correlation between mechanical stiffness and thermal transport along the solid framework of a uniaxially compressed polyurea aerogel. J. Non-Cryst. Solids.

[69] Gould G.L., Lee J.K., Stepanian C.J., Lee K.P. (2009). High Strength Nanoporous Bodies Reinforced with Fibrous Materials. U.S. Patent.

[70] Weigold L., Reichenauer G. (2015). Correlation between the elastic modulus and heat transport along the solid phase in highly porous materials: Theoretical approaches and experimental validation using polyurea aerogels. J. Supercrit. Fluids.

[71] Malakooti S., Churu H.G., Lee A., Xu T., Luo H., Xiang N., Sotiriou-Leventis C., Leventis N., Lu H. (2017). Sound insulation properties in low-density, mechanically strong and ductile nanoporous polyurea aerogels. J. Non-Cryst. Solids.

[72] Lu H., Xiang H., Leventis N., Sotiriou-Leventis C. (2015). Acoustic Attenuators Based on Porous Nanostructured Materials. U.S. Patent.

[73] Malakooti S., Gitogo C.H., Lee A., Rostami S., May S.J., Ghidei S., Wang F., Lu Q., Luo H., Xiang N. (2018). Sound transmission loss enhancement in an inorganic-organic laminated wall panel using multifunctional low-density nanoporous polyurea aerogels: Experiment and modeling. Adv. Eng. Mater..

[74] Price M.A., Aslam T.D., Quirk J.J. (2018). Analysis of steady compaction waves in polyurea aerogel. AIP Conf. Proc..

[75] Whitworth N., Lambourn B. (2018). A single-phase analytic equation of state for solid polyurea and polyurea aerogels. AIP Conf. Proc..

[76] Pacheco A.H., Gustavsen R.L., Aslam T.D., Bartram B.D. (2017). Hugoniot based equation of state for solid polyurea and polyurea aerogels. AIP Conf. Proc..

[77] Aslam T.D., Gustavsen R.L., Bartram B.D. (2014). An equation of state for polyurea aerogel based on multi-shock response. J. Phys. Conf. Series.

[78] Wu C., Taghvaee T., Wei C., Ghasemi A., Chen G., Leventis N., Gao W. (2018). Multi-scale progressive failure mechanism and mechanical properties of nanofibrous polyurea aerogels. Soft Matter.

[79] Fakharifar M., Lin Z., Wu C., Mahadik-Khanolkar S., Leventis N., Chen G. (2013). Microstructural characteristics of polyurea and polyurethane xerogels for concrete confinement with FRP system. Adv. Mater. Res..

[80] Li Y., Liao W., Taghvaee T., Wu C., Ma H., Leventis N. (2019). Bioinspired strong nanocellular composite prepared with magnesium phosphate cement and polyurea aerogel. Mater. Lett..

[81] Yin W., Lu H., Leventis N., Rubenstein D.A. (2013). Characterization of the Biocompatibility and Mechanical Properties of Polyurea Organic Aerogels with the Vascular System: Potential as a Blood Implantable Material. Int. J. Polym. Mater. Polym. Biomater..

[82] Gu S., Jana S.C. (2017). Open cell aerogel foams with hierarchical pore structures. Polymer.

[83] Taghvaee T., Donthula S., Rewatkar P.M., Majedi Far H., Sotiriou-Leventis C., Leventis N. (2019). *K*-index: Descriptor, predictor, and correlator of complex nanomorphology to other material properties. ACS Nano.

[84] Rege A., Preibisch I., Schestakow M., Ganesan K., Gurikov P., Milow B., Smirnova I., Itskov M. (2018). Correlating Synthesis Parameters to Morphological Entities: Predictive Modeling of Biopolymer Aerogels. Materials.

[85] Tripathi A., Parsons G.N., Khan S.A., Rojas O.J. (2018). Synthesis of organic aerogels with tailorable morphology and strength by controlled solvents swelling following Hansen solubility. Sci. Rep..

[86] Zhu Z., Snellings G.M.B.F., Koebel M.M., Malfait W.J. (2017). Superinsulating polyisocyanate based aerogels: A targeted search for the optimum solvent system. ACS Appl. Mater. Interfaces.

[87] Jenkins G.M., Kawamura K. (1976). Polymeric Carbons, Carbon Fibre, Glass and Char.

[88] McKenzie D.R., Muller D., Pailthorpe B.A. (1991). Compressive-stress-induced formation of thin-film tetrahedral amorphous carbon. Phys. Rev. Lett..

[89] Loelsberg W., Fricke M., Weinrich D., Leventis N., Sotiriou-Leventis C., Saeed A.M. (2020). Process for Producing Isocyanate-Based Xerogels and Aerogels with Mineral acids. U.S. Patent.

[90] Leventis N., Chidambareswarapattar C., Mohite D.P., Larimore Z.J., Lu H., C Sotiriou-Leventis C. (2011). Multifunctional porous aramids (aerogels) by efficient reaction of carboxylic acids and isocyanates. J. Mater. Chem..

[91] Leventis N., Sotiriou-Leventis C., Chidambareswarapattar C. (2014). Multifunctional Porous Aramids (Aerogels) and Fabrication Thereof. U.S. Patent.

[92] Leventis N., Sotiriou-Leventis C., Saeed M.A. (2016). Multifunctional Porous Aramids (Aerogels) and Fabrication Thereof. U.S. Patent.

[93] Leventis N., Sotiriou-Leventis C., Saeed M.A. (2017). Multifunctional Porous Aramids (Aerogels), Fabrication Thereof, and Catalytic Compositions Derived Therefrom. U.S. Patent.

[94] Saeed A.M., Wisner C.A., Donthula S., Majedi Far H., Sotiriou-Leventis C., Leventis N. (2016). Reuseable monolithic nanoporous graphite-supported nanocatalysts (Fe, Au, Pt, Pd, Ni, and Rh) from pyrolysis and galvanic transmetalation of ferrocene-based polyamide aerogels. Chem. Mater..

[95] Saeed A.M., Rewatkar P.M., Majedi Far H., Taghvaee T., Donthula S., Mandal C., Sotiriou-Leventis C., Leventis N. (2017). Selective CO_2_ sequestration with monolithic bimodal micro/macroporous carbon aerogels derived from stepwise pyrolytic decomposition of polyamide-polyimide-polyurea random co-polymers. ACS Appl. Mater. Interfaces.

[96] Leventis N., Sotiriou-Leventis C., Saeed M.A. (2021). Novel Porous Polymer Compositions for the Synthesis of Monolithic Bimodal Microporous/Macroporous Carbon Compositions Useful for Selective CO2 Sequestration. U.S. Patent.

[97] Chidambareswarapattar C., Larimore Z., Sotiriou-Leventis C., Mang J.T., Leventis N. (2010). One-step room-temperature synthesis of fibrous polyimide aerogels from anhydrides and isocyanates and conversion to isomorphic carbons. J. Mater. Chem..

[98] Chidambareswarapattar C., Sotiriou-Leventis C., Leventis N. (2010). One-step polyimide aerogels from anhydrides and isocyanates. Polym. Prepr..

[99] Chidambareswarapattar C., Xu L., Sotiriou-Leventis C., Leventis N. (2013). Robust monolithic multiscale nanoporous polyimides and conversion to isomorphic carbons. RSC Adv..

[100] Leventis N., Sotiriou-Leventis C., Chidambareswarapattar C. (2017). Porous Nanostructured Polyimide Networks and Methods of Manufacture. U.S. Patent.

[101] Majedi Far H., Rewatkar P.M., Donthula S., Taghvaee T., Saeed A.M., Sotiriou-Leventis C., Leventis N. (2019). Exceptionally high CO_2_ adsorption at 273 K by microporous carbons from phenolic aerogels: The role of heteroatoms in comparison with carbons from polybenzoxazine and other organic aerogels. Macromol. Chem. Phys..

[102] Zhang G., Dass A., Rawashdeh A.-M.M., Thomas J., Counsil J.A., Sotiriou-Leventis C., Fabrizio E.F., Ilhan F., Vassilaras P., Scheiman D.A. (2004). Isocyanate cross-linked silica aerogel monoliths: Preparation and characterization. J. Non-Cryst. Solids.

[103] Leventis N., Lu H., Aegerter M., Leventis N., Koebel M. (2011). Polymer Crosslinked Aerogels. Aerogels Handbook—Advances in Sol-Gel Derived Materials and Technologies.

[104] Rewatkar P.M., Taghvaee T., Saeed A.M., Donthula S., Mandal C., Chandrasekaran N., Leventis T., Shruthi T.K., Sotiriou-Leventis C., Leventis N. (2018). Sturdy, monolithic SiC and Si_3_N_4_ aerogels from compressed polymer-cross-linked silica xerogel powders. Chem. Mater..

[105] Katti A., Shimpi N., Roy S., Lu H., Fabrizio E.F., Dass A., Capadona L.A., Leventis N. (2006). Chemical, physical and mechanical characterization of isocyanate-crosslinked amine-modified silica aerogels. Chem. Mater..

[106] Mandal C., Donthula S., Majedi Far H., Saeed A.M., Sotiriou-Leventis C., Leventis N. (2019). Transparent, mechanically strong, thermally insulating cross-linked silica aerogels for energy-efficient windows. J. Sol-Gel Sci. Technol..

[107] Rewatkar P.M., Soni R.U., Sotiriou-Leventis C., Leventis N. (2019). A cobalt sunrise: Thermites based on LiClO_4_-filled Co(0) aerogels prepared from polymer-cross-linked cobaltia xerogel powders. ACS Appl. Mater. Interfaces.

[108] Paraskevopoulou P., Smirnova I., Athamneh T., Papastergiou M., Chriti D., Mali G., Čendak T., Chatzichristidi M., Raptopoulos G., Gurikov P. (2020). Mechanically strong polyurea/polyurethane-cross-linked alginate aerogels. ACS Appl. Polym. Mater..

[109] Paraskevopoulou P., Smirnova I., Athamneh T., Papastergiou M., Chriti D., Mali G., Čendak T., Raptopoulos G., Gurikov P. (2020). Polyurea-crosslinked biopolymer aerogel beads. RSC Adv..

[110] Paraskevopoulou P., Raptopoulos G., Len A., Dudás Z., Fábián I., Kalmár J. (2021). Fundamental skeletal nanostructure of nanoporous polymer-cross-linked alginate aerogels and its relevance to environmental remediation. ACS Appl. Nano Mater..

[111] Paraskevopoulou P., Raptopoulos G., Leontaridou F., Papastergiou M., Sakellari A., Karavoltsos S. (2021). Evaluation of polyurea-crosslinked alginate aerogels for seawater decontamination. Gels.

[112] Georgiou E., Raptopoulos G., Papastergiou M., Paraskevopoulou P., Pashalidis I. (2022). Extremely efficient uranium removal from aqueous environments with polyurea-crosslinked alginate aerogel beads. ACS Appl. Polym. Mater..

[113] Raptopoulos G., Papastergiou M., Chriti D., Effraimopoulou E., Čendak T., Samartzis N., Mali G., Ioannides T., Gurikov P., Smirnova I. (2021). Metal-doped carbons from polyurea-crosslinked alginate aerogel beads. Mater. Adv..

[114] Wang P., Ma X., Li Q., Yang B., Shang J., Deng Y. (2016). Green synthesis of polyureas from CO_2_ and diamines with a functional ionic liquid as the catalyst. RSC Adv..

[115] Qaroush A., Al-Hamayda A.S., Khashman Y.K., Vagin S. (2013). Highly efficient isocyanate-free microwave-assited synthesis of [6]-oligourea. Catal. Sci. Technol..

[116] Tiwari L., Kumar V., Kumar B., Majajan D. (2018). A practiclly simple, catalyst free and scalablesynthesis of *N*-substitutedureas in water. RSC Adv..

